# Ionic current changes underlying action potential repolarization responses to physiological pacing and adrenergic stimulation in adult rat ventricular myocytes

**DOI:** 10.14814/phy2.15766

**Published:** 2023-07-26

**Authors:** Luke A. Howlett, Harley Stevenson‐Cocks, Michael A. Colman, Matthew K. Lancaster, Alan P. Benson

**Affiliations:** ^1^ Faculty of Biological Sciences University of Leeds Leeds UK; ^2^ Faculty of Medical Sciences Newcastle University Newcastle upon Tyne UK

**Keywords:** action potential, computational modeling, electrophysiology, exercise, ion currents, repolarization

## Abstract

This study aimed to simulate ventricular responses to elevations in myocyte pacing and adrenergic stimulation using a novel electrophysiological rat model and investigate ion channel responses underlying action potential (AP) modulations. Peak ion currents and AP repolarization to 50% and 90% of full repolarization (APD_50‐90_) were recorded during simulations at 1–10 Hz pacing under control and adrenergic stimulation conditions. Further simulations were performed with incremental ion current block (L‐type calcium current, I_Ca_; transient outward current, I_to_; slow delayed rectifier potassium current, I_Ks_; rapid delayed rectifier potassium current, I_Kr_; inward rectifier potassium current, I_K1_) to identify current influence on AP response to exercise. Simulated APD_50‐90_ closely resembled experimental findings. Rate‐dependent increases in I_Ks_ (6%–101%), I_Kr_ (141%–1339%), and I_Ca_ (0%–15%) and reductions in I_to_ (11%–57%) and I_K1_ (1%–9%) were observed. Meanwhile, adrenergic stimulation triggered moderate increases in all currents (23%–67%) except I_K1_. Further analyses suggest AP plateau is most sensitive to modulations in I_to_ and I_Ca_ while late repolarization is most sensitive to I_K1_, I_Ca_, and I_Ks_, with alterations in I_Ks_ predominantly stimulating the greatest magnitude of influence on late repolarization (35%–846% APD_90_ prolongation). The modified Leeds rat model (mLR) is capable of accurately modeling APs during physiological stress. This study highlights the importance of I_Ca_, I_to_, I_K1,_ and I_Ks_ in controlling electrophysiological responses to exercise. This work will benefit the study of cardiac dysfunction, arrythmia, and disease, though future physiologically relevant experimental studies and model development are required.

## 1 INTRODUCTION

The rat is an extensively‐used animal model in experimental cardiac electrophysiology studies (Hardy et al., 2018; Janczewski et al., 2002; Josephson et al., 2002; Liu et al., 2000; Sakatani et al., 2006; Walker et al., 1993; Wang & Fitts, 2017; Wang & Fitts, 2020). However, existing investigations relating to cardiac excitation and conduction in the adult rat have predominantly evaluated changes in myocyte action potential (AP) duration (APD) and AP repolarization under non‐physiologically relevant conditions, performed often at non‐physiologically relevant pacing frequencies (Banyasz et al., 2014; Bao et al., 2013; Cerbai et al., 1994; Farrell & Howlett, 2007; Gan et al., 2013; Kang et al., 2017; Liu et al., 2000; Natali et al., 2002; Sala et al., 2017). The lack of information regarding changes in rat ventricular AP repolarization to physiological stress such as changes in pacing has, in part, been responsible for the lack of available rat myocyte‐based computational models (that can be used as an additional research tool to understand mechanisms underlying experimental phenomena) capable of accurately and reliably modeling APD changes at a whole range of physiologically relevant pacing frequencies as would be observed in the whole‐heart during exercise. A similar lack of experimental investigation focusing on the impact of isoproterenol at such pacing frequencies (Kang et al., 2017; Sala et al., 2017; Stuart et al., 2018; Wang & Fitts, 2017) has also impaired the generation of computational models capable of modeling a physiologically relevant adrenergic response which helps define the exercise response as would occur in vivo.

The reliable and accurate modeling of APD changes in the rat heart to typical physiological stress such as increased pacing and adrenergic stimulation as a result of physical activity and exercise is vital in order to improve our understanding of the underlying ion channel changes facilitating such dynamic responses experienced daily by most humans, and will help to not only expedite the potential identification of therapeutic strategies through isolating key players in exercise response within excitation but also provide a substantial reference for comparison to and investigation of diseased and aged hearts.

Computational rat models by Pandit et al. (2001) and Gattoni et al. (2016) have partly addressed this problem through their capacity to model APD at pacing frequencies somewhat equivalent to the resting rat heart rate under physiological temperatures. However, these models are unable to model APD at greater pacing frequencies (Gattoni et al., 2016; Pandit et al., 2001).

Recently, a model capable of simulating changes in rat ventricular myocyte electrophysiology and calcium handling at greater pacing frequencies based on previous works by Gattoni et al. (2016) and Colman et al. (2017, 2019) has been developed in our laboratory by Stevenson‐Cocks et al., named the Leeds rat (LR) model (Stevenson‐Cocks, 2019). More recently, the LR model has been further developed through the comparison against experimental APD and ion channel findings in adult rat ventricular myocytes from our laboratory (Howlett, 2022; Howlett et al., 2022) and in turn is described as the modified LR (mLR) model throughout the remainder of this manuscript (Howlett, 2022). The mLR model is capable of accurately modeling APD at a range of physiological pacing frequencies under control and adrenergic stimulation conditions (Howlett, 2022). The mLR model also provides adequate comparison to ion channel data recorded at low frequencies (Howlett, 2022). However, as a consequence of limited ion current investigation at elevated pacing frequencies (Bébarová et al., 2014; Fauconnier et al., 2005; He et al., 2008; Jourdon & Feuvray, 1993; Kilborn & Fedida, 1990; Liu et al., 2000; Meszaros et al., 1997; Sakatani et al., 2006; Scamps et al., 1990; Vizgirda et al., 2002; Wahler, 1992; Walker et al., 1993; Wang & Fitts, 2020; Xiao et al., 1994; Zhou et al., 1998), very little is known about ion channel function response to strenuous exercise and, in turn, the changes that underlie and mediate the subsequent response in AP repolarization in the rat ventricle.

The aim of this study was to use the mLR model to simulate AP and ion channel currents at various pacing frequencies between 1 and 10 Hz under control and adrenergic stimulation conditions (isoproterenol superfusion) and compare mLR model simulations with data from laboratory experiments and original LR model simulations, and further, to perform a sensitivity analysis in the mLR model to provide insight on the ionic changes underlying APD modulations and identify which ion channels have the greatest influence on APD response to emulated exercise.

## 2 RESULTS

### 2.1 Modeling action potential repolarization responses to changes in pacing and adrenergic stimulation

**FIGURE 1:**
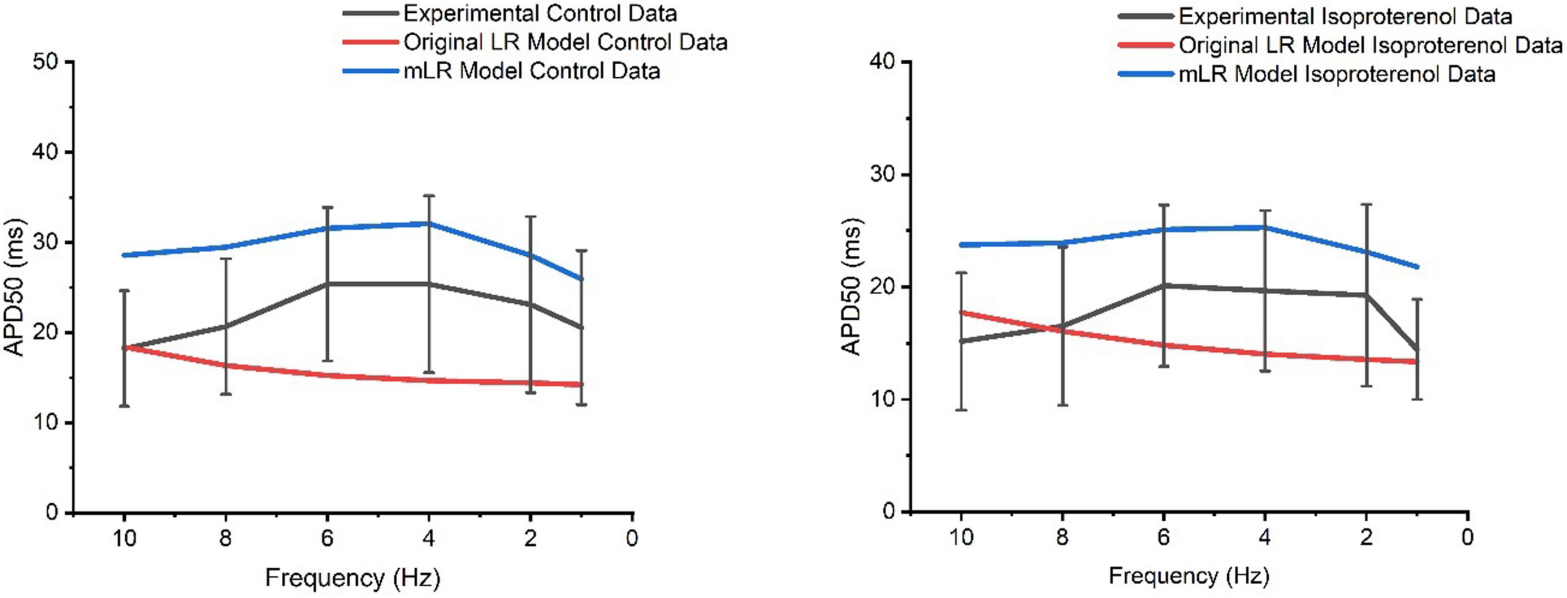
APD_50_ data simulated by the mLR model (Howlett, 2022) and original LR model (Stevenson‐Cocks, 2019) compared with APD_50_ laboratory data (Howlett et al., 2022) during control and adrenergic stimulation conditions. Experimental data presented as mean with 95% confidence interval (1.96 × SEM) error bars. Experimental data based on *N* = 8. Experimental isoproterenol data based on findings from 5 nM isoproterenol dose. Figure adapted from Howlett (2022).

**FIGURE 2:**
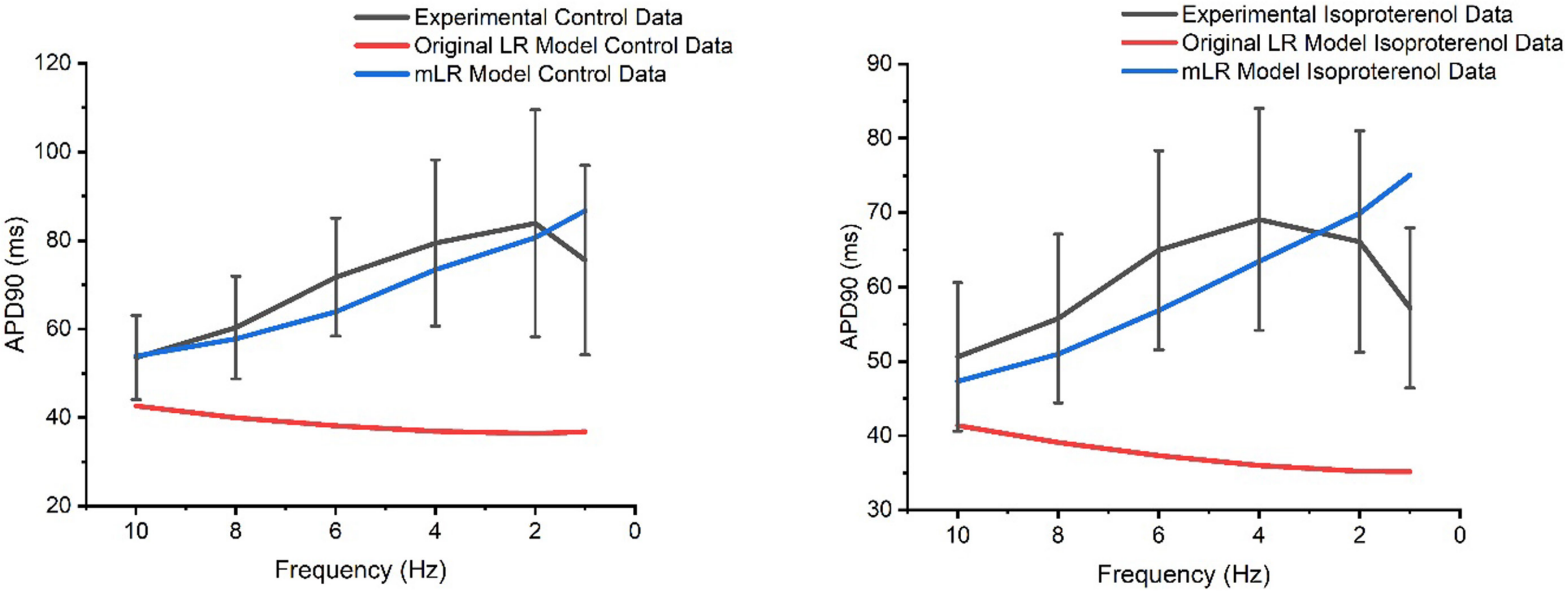
APD_90_ data simulated by the mLR model (Howlett, 2022) and original LR model (Stevenson‐Cocks, 2019) compared with APD_90_ laboratory data (Howlett et al., 2022) during control and adrenergic stimulation conditions. Experimental data presented as mean with 95% confidence interval (1.96 × SEM) error bars. Experimental data based on *N* = 8. Experimental isoproterenol data based on findings from 5 nM isoproterenol dose. Figure adapted from Howlett (2022).

**FIGURE 3:**
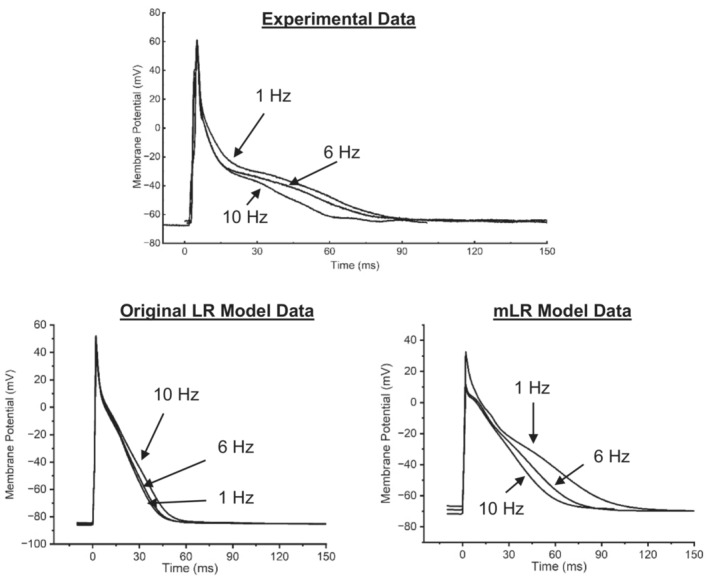
Example action potential traces from experimental data (Howlett et al., 2022) and simulated AP traces from the original LR model (Stevenson‐Cocks, 2019) and the mLR model (Howlett, 2022). Traces recorded at 1, 6, and 10 Hz pacing as displayed. Figure adapted from Howlett (2022), Howlett et al. (2022), and Stevenson‐Cocks (2019).

Figures 1, 2, 3 display APD_50‐90_ responses to varied pacing and adrenergic stimulation in rat ventricular myocytes during laboratory investigations (Howlett et al., 2022) and also during original LR model and mLR model simulations. During control conditions (Figures 1, 2, 3), the mLR model simulated APD_50_ and APD_90_ within the 95% confidence interval of laboratory data (Howlett et al., 2022) at 1–6 Hz pacing and 1–10 Hz pacing, respectively. Meanwhile, the original LR model simulated APD_50_ within the 95% confidence interval of laboratory data (Howlett et al., 2022) at 1, 2, 8, and 10 Hz pacing, but failed to simulate APD_90_ within the 95% confidence interval of laboratory data (Howlett et al., 2022) at any pacing frequency. During adrenergic stimulation conditions, the mLR model simulated APD_50_ and APD_90_ within the 95% confidence interval of laboratory data (Howlett et al., 2022) at 2–6 Hz pacing and 2–10 Hz pacing, respectively. The mLR model also simulated relative adrenergic responses in APD_50_ and APD_90_ within the 95% confidence interval of laboratory data (Howlett et al., 2022) at 2–10 Hz pacing and 2–8 Hz pacing, respectively. Conversely, during adrenergic stimulation, the original LR model simulated APD_50_ and APD_90_ within the 95% confidence interval of laboratory data at 1–10 Hz pacing and 10 Hz pacing only, respectively. In addition, the original LR model simulated relative adrenergic responses in APD_50_ and APD_90_ within the 95% confidence interval of laboratory data at 2 Hz pacing and 4–10 Hz pacing, respectively.

Overall, simulations by the original LR model and mLR model demonstrated APD_50_ values that were relatively similar to APD_50_ values from experimental recordings during control and adrenergic stimulation conditions at a range of pacing frequencies. Conversely, simulations by the mLR model demonstrated APD_90_ values similar to APD_90_ values from experimental recordings during control and adrenergic stimulation conditions at a range of pacing frequencies to a much greater extent compared with APD_90_ values from original LR model simulations, suggesting the mLR model is capable (at least qualitatively) of accurately simulating APD_50‐90_ in the adult rat during varied physiologically relevant stress.

**FIGURE 4:**
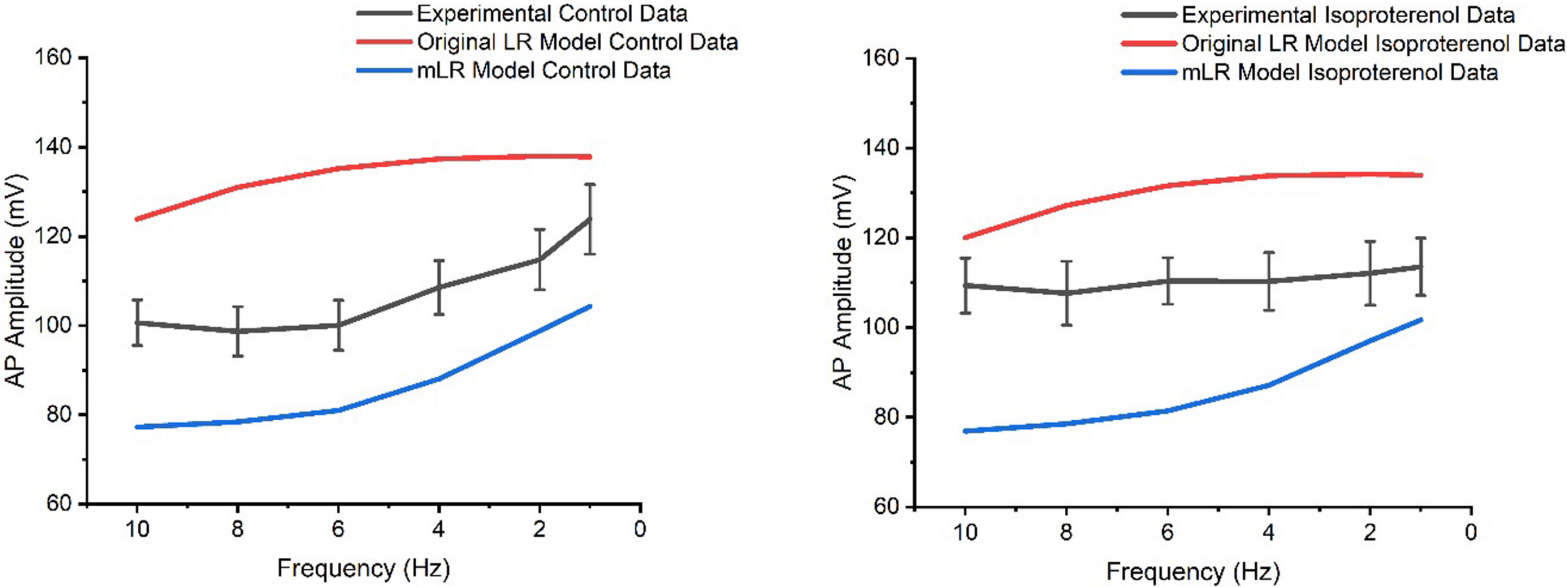
AP amplitude simulated by the mLR model (Howlett, 2022) and original LR model (Stevenson‐Cocks, 2019) compared with laboratory data (Howlett et al., 2022) during control and adrenergic stimulation conditions. Experimental data presented as mean with 95% confidence interval (1.96 × SEM) error bars. Experimental data based on *N* = 8. Experimental isoproterenol data based on findings from 5 nM isoproterenol dose. Figure adapted from Howlett (2022).

**FIGURE 5:**
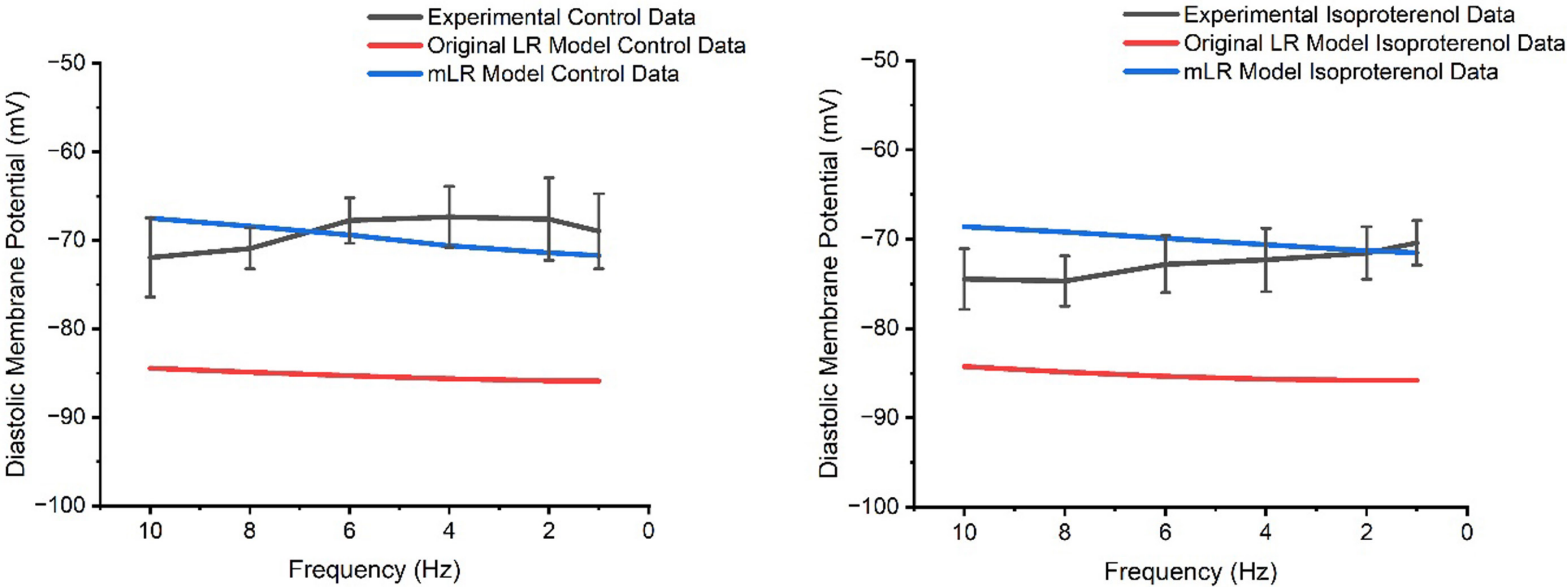
Diastolic membrane potential simulated by the mLR model (Howlett, 2022) and original LR model (Stevenson‐Cocks, 2019) compared with laboratory data (Howlett et al., 2022) during control and adrenergic stimulation conditions. Experimental data presented as mean with 95% confidence interval (1.96 × SEM) error bars. Experimental data based on *N* = 8. Experimental isoproterenol data based on findings from 5 nM isoproterenol dose. Figure adapted from Howlett (2022).

Figures 4 and 5 display AP amplitude and diastolic membrane potential responses to varied pacing and adrenergic stimulation in rat ventricular myocytes during laboratory investigations (Howlett et al., 2022) and simulations by the original LR model and mLR model. Figure 4 shows during both control and adrenergic stimulation conditions, the original LR model and the mLR model were unable to simulate AP amplitude data within the 95% confidence interval of the experimental data at any pacing frequency. However, reductions in AP amplitude in response to increased pacing were similarly observed in experimental data and model simulations and though outside the 95% confidence interval of experimental data, rate‐dependent AP amplitude changes simulated by the mLR model during control conditions appear to more closely resemble rate‐dependent AP amplitude changes in experimental data compared with original LR model simulations. Furthermore, in response to adrenergic stimulation, AP amplitude simulated by the original LR model and mLR model similarly demonstrated minor reductions compared with control conditions, while AP amplitude increased at low pacing frequencies and reduced at high pacing frequencies in response to adrenergic stimulation compared with control conditions in the experimental data. Conversely, the mLR model simulated diastolic membrane potential within the 95% confidence interval of the experimental data at 1, 2, 4, 6, and 10 Hz pacing frequencies during control conditions and at 1–6 Hz pacing during adrenergic stimulation. Meanwhile, the original LR was unable to simulate diastolic membrane potential within the 95% confidence interval of experimental data at any pacing frequency.

### 2.2 Modeling ion channel responses to changes in pacing and adrenergic stimulation

We have shown that the mLR model reproduces experimental APD restitution (Howlett et al., 2022); now, we use the model to identify which currents are responsible for the observed APD restitution responses under control and adrenergic stimulation conditions.

**FIGURE 6:**
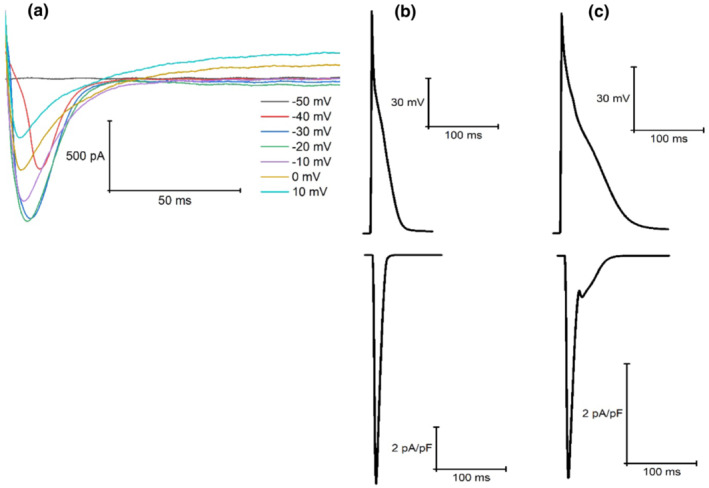
Example steady‐state I_Ca_ current races recorded from laboratory experiments (a) (Howlett et al., 2022) and simulations from the original LR model (b) (Stevenson‐Cocks, 2019) and the mLR model (c) (Howlett, 2022). All data recorded at 1 Hz. Experimental data based on voltage‐clamp recordings. Figure adapted from Howlett (2022).

**FIGURE 7:**
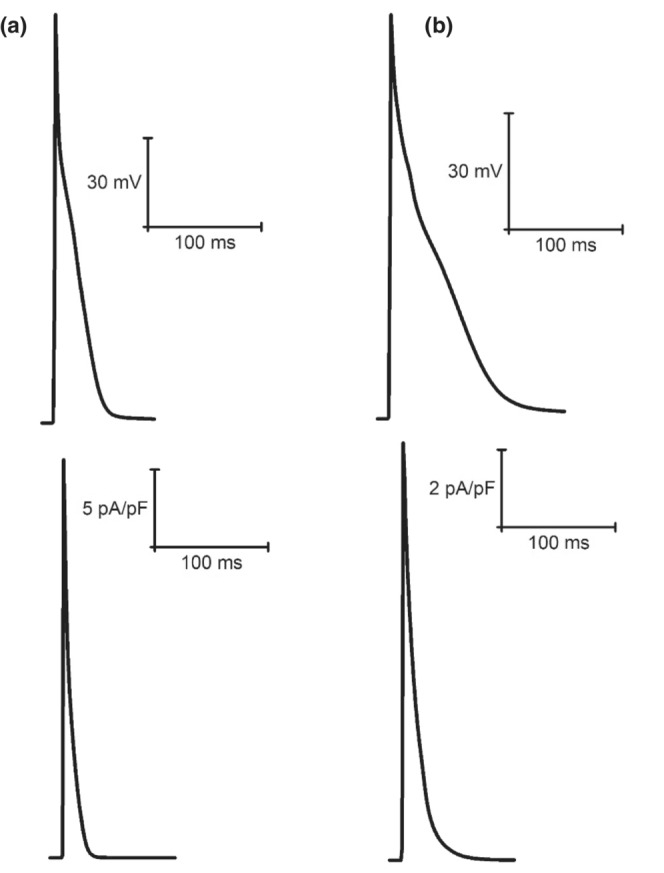
Example steady‐state I_to_ current traces recorded from original LR model (a) (Stevenson‐Cocks, 2019) and mLR model (b) simulations (Howlett, 2022). All data recorded at 1 Hz.

**FIGURE 8:**
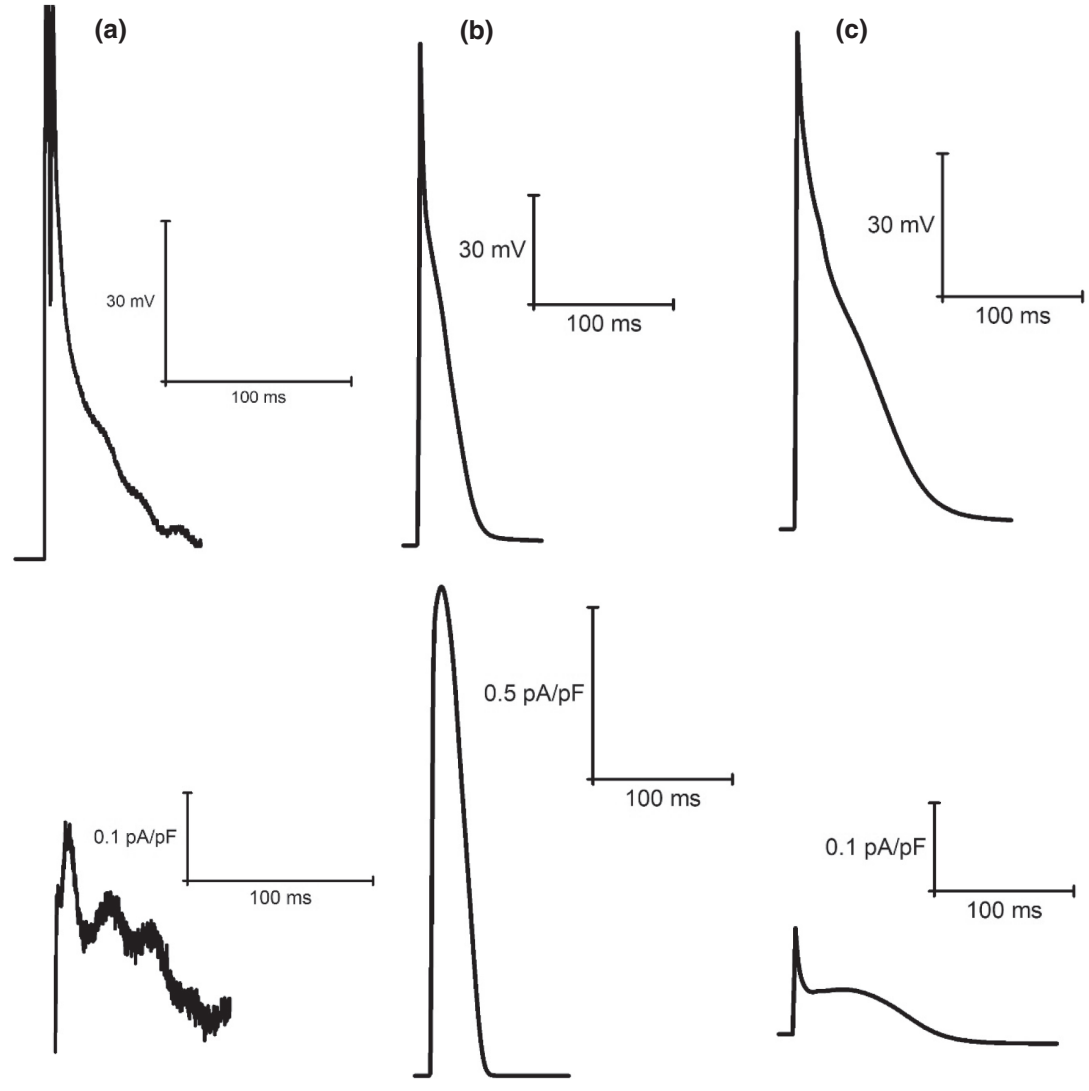
Example steady‐state I_Kr_ current traces recorded from laboratory experiments (a) (Howlett et al., 2022) and simulations from the original LR model (b) (Stevenson‐Cocks, 2019) and the mLR model (c) (Howlett, 2022). All data recorded at 1 Hz. Experimental data based on E4031‐sensitive current recordings, assumed to reflect I_Kr_ current. Original LR model I_ss_ data were used to provide comparison with mLR model and experimental I_Kr_ data. Figure adapted from Howlett (2022).

**FIGURE 9:**
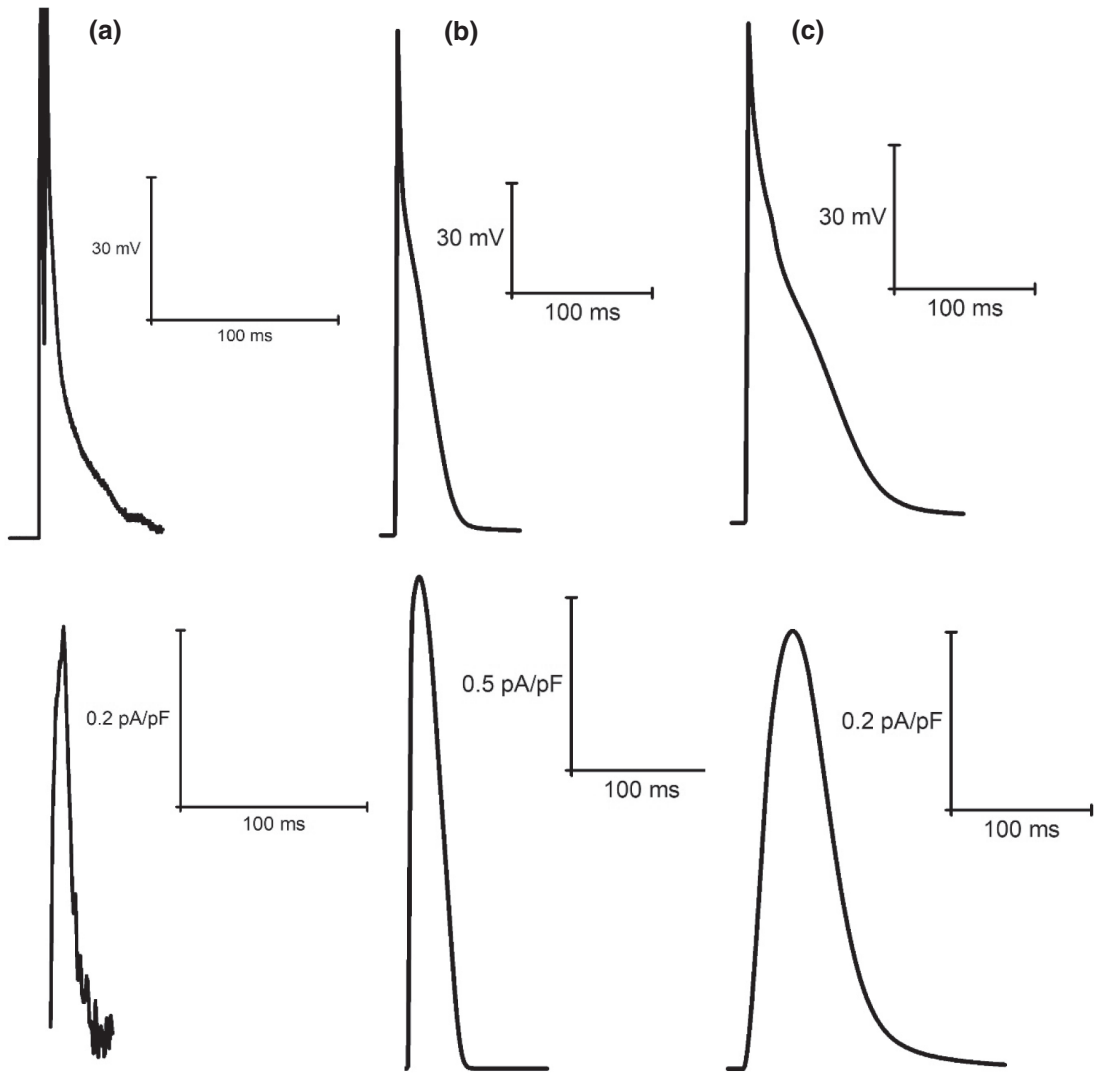
Example steady‐state I_Ks_ current traces recorded from laboratory experiments (a) (Howlett et al., 2022) and simulations from the original LR model (b) (Stevenson‐Cocks, 2019) and the mLR model (c) (Howlett, 2022). All data recorded at 1 Hz. Experimental data based on chromanol 293b‐sensitive current recordings, assumed to reflect I_Ks_ current. Original LR model I_ss_ data were used to provide comparison with mLR model and experimental I_Ks_ data. Figure adapted from Howlett (2022).

**FIGURE 10:**
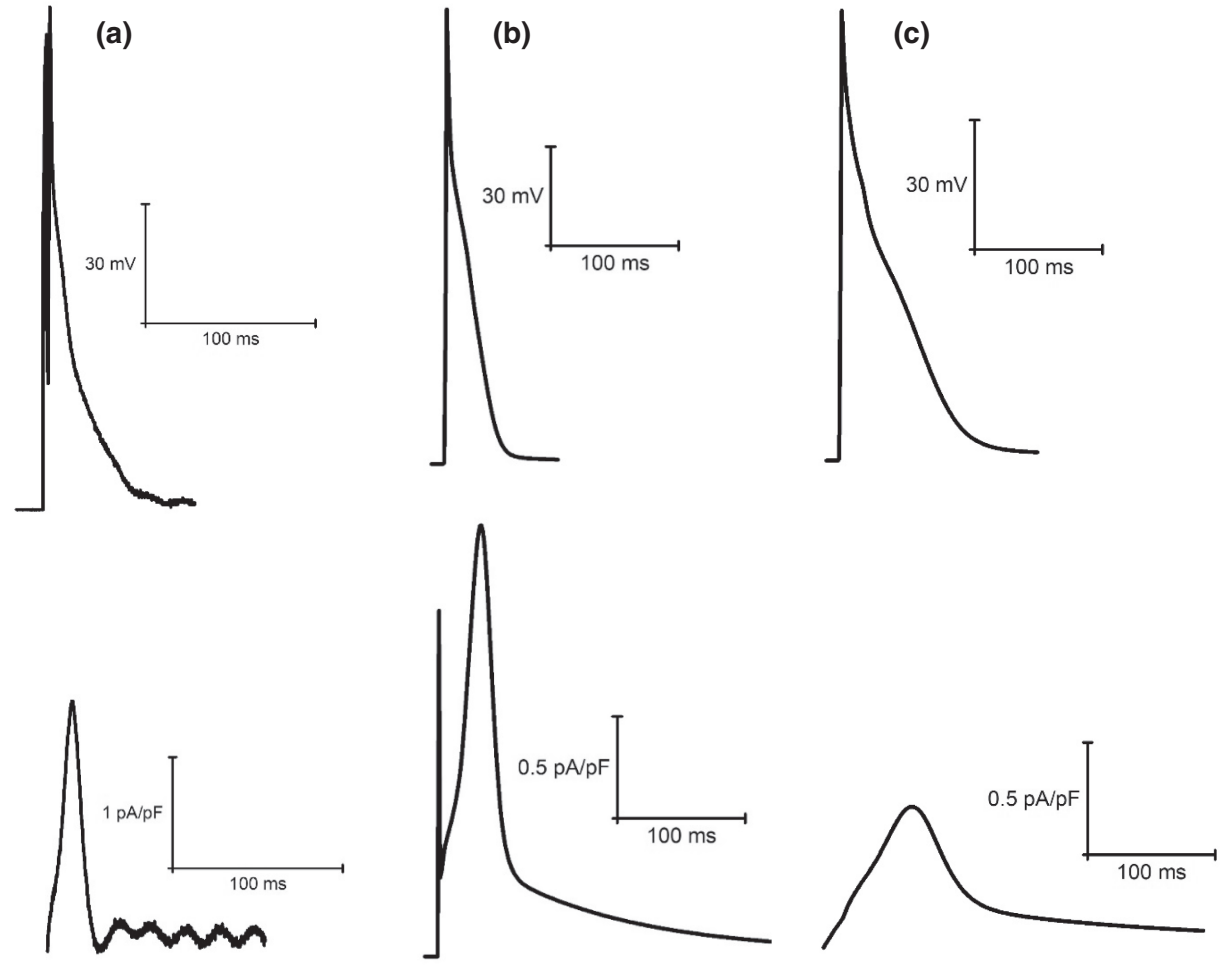
Example steady‐state I_K1_ current traces recorded from laboratory experiments (a) (Howlett et al., 2022) and simulations from the original LR model (b) (Stevenson‐Cocks, 2019) and the mLR model (c) (Howlett, 2022). All data recorded at 1 Hz. Experimental data based on BaCl_2_‐sensitive current recordings, assumed to reflect I_K1_ current. Figure adapted from Howlett (2022).

**FIGURE 11:**
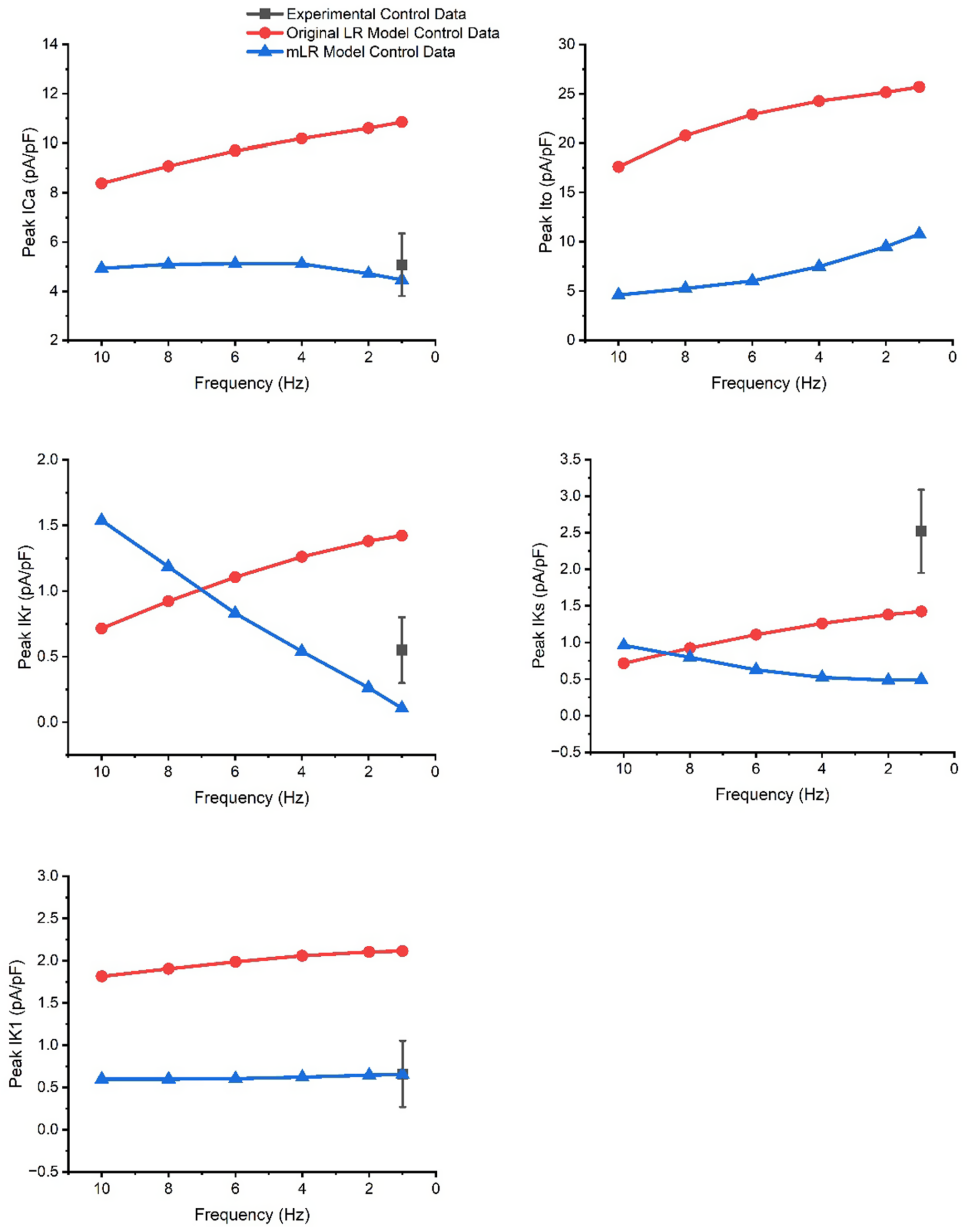
Peak I_Ca_, I_to_, I_Kr_, I_Ks,_ and I_K1_ current simulated by the original LR model and the mLR model at 1–10 Hz during control conditions. Where possible, simulated peak ion current data were compared with experimental data recorded at 1 Hz from our laboratory (Howlett et al., 2022). Experimental data presented as mean with 95% confidence interval (1.96 × SEM) error bars. Experimental data based on *N* = 6 (peak I_Ca_) and *N* = 8 (peak I_Kr_, I_Ks,_ and I_K1_). Original LR model I_ss_ data were plotted to provide comparison with mLR model and experimental I_Ks_ and I_Kr_ data. Figure adapted from Howlett (2022).

**FIGURE 12:**
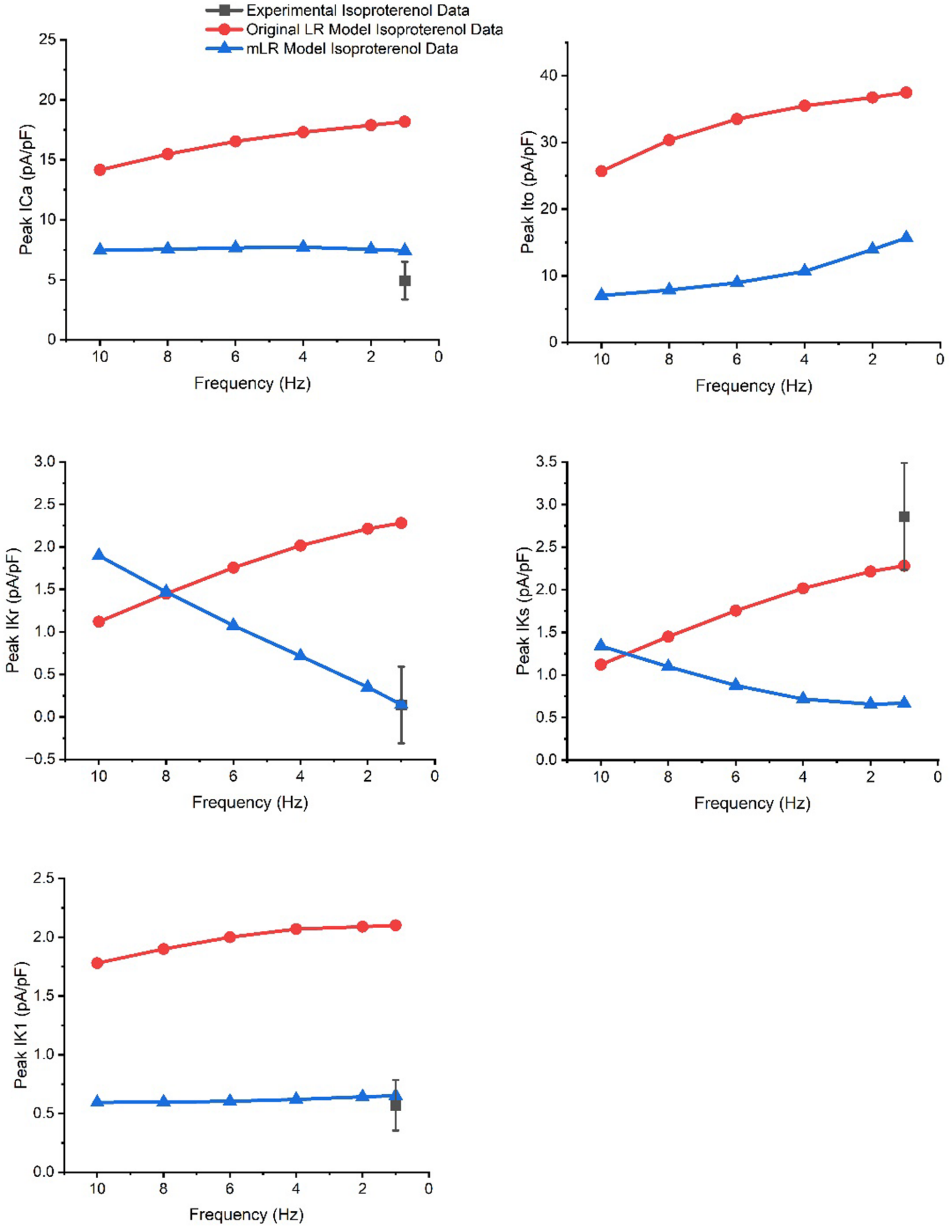
Peak I_Ca_, I_to_, I_Kr_, I_Ks,_ and I_K1_ current simulated by the original LR model and the mLR model at 1–10 Hz during adrenergic stimulation. Where possible, simulated peak ion current data were compared with experimental data recorded at 1 Hz from our laboratory during 5 nM isoproterenol superfusion (Howlett et al., 2022). Experimental data presented as mean with 95% confidence interval (1.96 × SEM) error bars. Experimental data based on *N* = 6 (peak I_Ca_) and *N* = 8 (peak I_Kr_, I_Ks,_ and I_K1_). Original LR model I_ss_ data were plotted to provide comparison with mLR model and experimental I_Ks_ and I_Kr_ data. Figure adapted from Howlett (2022).

When simulated peak current data (at 1 Hz pacing) were compared with available experimental findings from our laboratory (Howlett, 2022) (Figures 6, 7, 8, 9, 10, 11, 12), I_Ca_ (4.46 vs. 5.08 ± 1.20 pA/pF; mean ± SD) and I_K1_ (0.66 vs. 0.66 ± 0.57 pA/pF) simulated by the mLR model during control conditions, alongside the I_K1_ (as a percent change from control: −1% vs. −14 ± 96%) and I_Kr_ (35% vs. −74 ± 463%) response to adrenergic stimulation simulated by the mLR model, remained within the 95% confidence interval of experimental findings (Figures 6, 8, 10 and 12). The adrenergic response in peak I_Ca_ simulated by the mLR model was far greater than experimental findings (67% vs −3 ± 22%) (Howlett, 2022). In addition, while peak I_Ks_ and subsequent response to adrenergic stimulation simulated by the mLR model did not remain within the 95% confidence interval of the experimental data, the magnitude of peak current response was relatively similar to findings from our laboratory investigations (35% vs. 13 ± 27%) (Howlett, 2022) (Figures 9, 11 and 12). With the exception of peak I_Ks_ during adrenergic stimulation, the original LR model was unable to simulate any peak current data within the 95% confidence interval of experimental data, frequently overestimating I_to_, I_Ca_, I_Kr,_ and I_K1_ (Figures 6, 7, 8 and 10, 11, 12). However, it is important to note that in Figures 11 and 12, simulated I_ss_ (due to the absence of I_Kr_ and I_Ks_) was used to compare original LR model data to experimental I_Ks_.

Figures 11 and 12 show peak I_Ca_ simulated by the mLR model increased steadily as pacing frequency was elevated, peaking at 6 Hz (15% increase vs. 1 Hz), while peak I_Ks_ (95% increase at 10 Hz vs. 1 Hz) and I_Kr_ (1339% increase at 10 Hz vs. 1 Hz) simulated by the mLR model increased to a greater extent at pacing frequencies ≥4 Hz and peak I_to_ (current reduced 57% at 10 Hz vs. 1 Hz) and I_K1_ simulated by the mLR model (9% reduction at 8 Hz vs. 1 Hz) declined continuously as pacing increased. Simulations by the original LR model showed I_Ca_, I_to_, I_kr_, I_Ks,_ and I_K1_ steadily reduced as pacing frequency was increased under control and adrenergic stimulation conditions (Figures 11 and 12). In response to adrenergic stimulation, I_Ca_, I_to,_ and I_ss_ simulated by the original LR model increased by magnitudes of 68%–71%, 46% and 57%–60% compared with control conditions, respectively, with responses remaining similar across all pacing frequencies. Furthermore, Figures 11 and 12 show the magnitude of I_Ca_ response to adrenergic stimulation simulated by the mLR model was a 48%–67% increase and was greatest at 1 Hz and lowest at 8 Hz. A similar magnitude of response was observed in I_to_ simulated by the mLR model (43%–53%) with the greatest response yielded at 10 Hz and the smallest response at 4 Hz. Adrenergic stimulation triggered increases of 23%–35% and 35%–40% magnitude, peaking at 10 Hz and 6 Hz in I_Kr_ and I_Ks_ simulated by the mLR model, respectively, compared with 1 Hz pacing. Almost no response was observed in I_K1_ (−1%) simulated by the mLR model or I_K1_ simulated by the original LR model (−2%–1%).

### 2.3 mLR model sensitivity analysis

#### 2.3.1 Ion current modulations underlying AP plateau (APD_50_ ) and late repolarization (APD_90_ ) response to changes in pacing and adrenergic stimulation in mLR model simulations

Table 1 shows I_to_ and I_Ca_ required the lowest magnitude of ion flux block (10%–20%) to stimulate a 5% change in APD_50_ at most pacing frequencies (1–6 Hz) during control conditions. Similarly, I_to_ and I_Ca_ required the lowest block (15%–35% and 25%–45%, respectively) to initiate a 5% change in APD_50_ at almost all pacing frequencies (1, 4–10 Hz) during adrenergic stimulation. During control conditions, however, at the greatest pacing frequencies (8–10 Hz), AP plateau was most sensitive to reductions in I_to_ and I_Ks_, evidenced by a 5% change in APD_50_ caused by flux block of only 10%–15% and 20%–25% magnitude, respectively (Table 1). At most pacing frequencies (1–4, 10 Hz), I_Kr_ required the greatest magnitude of block (> 40%) to stimulate change in AP plateau (APD_50_) during control conditions and also required the greatest block (125%–175%) during adrenergic stimulation before changing APD_50_ by at least 5%. The lack of APD_50_ sensitivity to reductions in I_Kr_ was especially evident at low pacing frequencies (1–4 Hz) as total block failed to trigger a minimum of 5% change in APD_50_ during both control and adrenergic stimulation conditions, with often no change present at all (Table 1).

**TABLE 1:** Percentage ion channel flux block required to stimulate a 5% change in APD_50_ during control (Con) and adrenergic stimulation (ISO) conditions simulated by the mLR model.

	I_Ca_		I_to_		I_Kr_		I_Ks_		I_K1_	
	Con	ISO	Con	ISO	Con	ISO	Con	ISO	Con	ISO
1 Hz	15%	25%	10%	15%	—	—	25%	70%	15%	25%
2 Hz	10%	25%	10%	15%	—	—	20%	60%	15%	20%
4 Hz	15%	25%	10%	15%	—	—	15%	45%	15%	30%
6 Hz	20%	30%	15%	20%	60%	175%	20%	45%	70%	90%
8 Hz	30%	40%	15%	25%	50%	125%	20%	45%	60%	90%
10 Hz	30%	45%	20%	35%	45%	—	25%	60%	40%	60%

Table 2 shows I_K1_ and I_Ca_ were the most sensitive markers of change in AP late repolarization (APD_90_) during low frequencies (<4 Hz) during control conditions. Ion channel flux block at magnitudes of 15% and 10% in I_Ca_ and I_K1,_ respectively, were sufficient to stimulate a 5% change in APD_90_. At greater pacing frequencies (6–8 Hz), I_K1_ and I_Ks_ required the lowest magnitude of current block (15% and 20%–25%, respectively) to produce change in late repolarization during control conditions, while at the most strenuous pacing frequency (10 Hz), APD_90_ were most sensitive to reductions in I_Ks_ current. On the contrary, I_Kr_ required the greatest block (50%–80%) at most pacing frequencies (1–6, 10 Hz) to alter APD_90_ by 5% during control conditions. Similarly, during adrenergic stimulation, AP late repolarization was least influenced by reductions in I_Kr_, evidenced through the requirement of a block of at least 125% to instigate a minimum of 5% change in APD_90_ at 4–10 Hz pacing, with total block failing to yield the minimum change in APD_90_ at the lowest pacing frequencies (1–2 Hz). Meanwhile, during adrenergic stimulation, I_K1_ and I_Ks_ required the least block (10%–30% and 25%–45%, respectively) to stimulate a 5% change in AP late repolarization.

**TABLE 2:** Percentage ion channel flux block required to stimulate a 5% change in APD_90_ during control (Con) and adrenergic stimulation (ISO) conditions simulated by the mLR model.

	I_Ca_		I_to_		I_Kr_		I_Ks_		I_K1_	
	Con	ISO	Con	ISO	Con	ISO	Con	ISO	Con	ISO
1 Hz	15%	40%	30%	80%	—	—	15%	25%	10%	10%
2 Hz	15%	35%	25%	100%	—	—	15%	30%	10%	10%
4 Hz	15%	40%	40%	125%	80%	125%	15%	35%	10%	10%
6 Hz	80%	60%	30%	125%	60%	125%	20%	40%	15%	15%
8 Hz	25%	70%	30%	125%	60%	125%	25%	40%	15%	20%
10 Hz	25%	60%	30%	150%	50%	150%	25%	45%	35%	30%

Overall, the results displayed a tendency, in all ion channels, to require a greater current block at higher pacing frequencies to yield the same or similar changes in APD_50‐90_ as produced at lower pacing frequencies during control and adrenergic stimulation conditions. Further, the results indicate that APD_50‐90_ changes are more resistant to ion current block during adrenergic stimulation, suggested by the greater block frequently required to provide a 5% change in AP plateau and late repolarization. Though for currents scaled up to mimic adrenergic stimulation (I_to_, I_Ca_, I_Kr_ and I_Ks_), this resistance may be offset by the greater current flux starting point/flux range.

**FIGURE 13:**
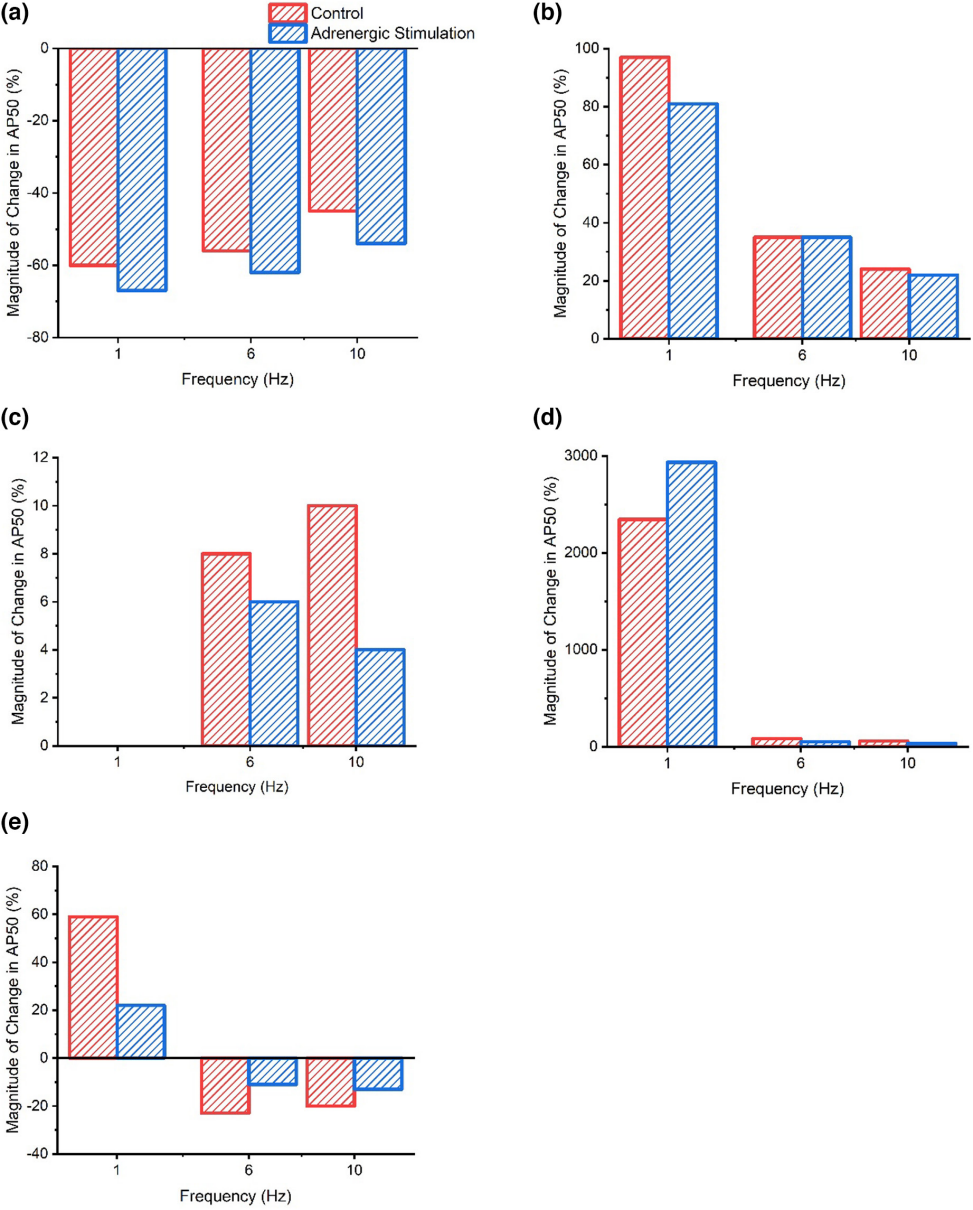
Peak magnitude of APD_50_ change during control and adrenergic stimulation conditions as a result of I_Ca_ (a), I_to_ (b), I_Kr_ (c), I_Ks_ (d), and I_K1_ (e) current flux block.

**FIGURE 14:**
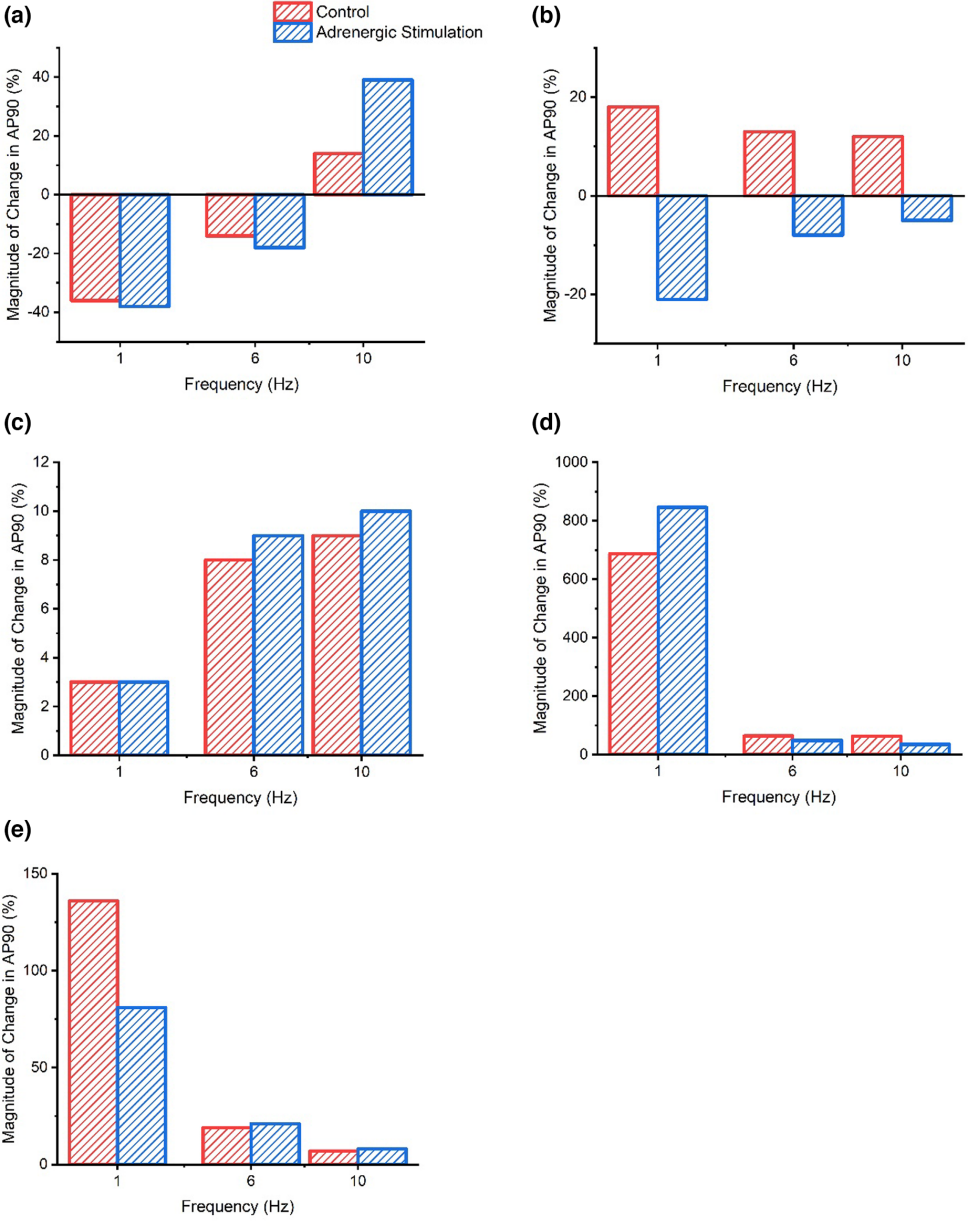
Peak magnitude of APD_90_ change during control and adrenergic stimulation conditions as a result of I_Ca_ (a), I_to_ (b), I_Kr_ (c), I_Ks_ (d), and I_K1_ (e) current flux block.

Figure 13 shows reductions in I_Ks_ had the greatest impact on AP plateau, prolonging APD_50_ by magnitudes between 2345% and 58% during control conditions, with the magnitude of change declining as pacing frequency increased. Similarly, during adrenergic stimulation, I_Ks_ block triggered the greatest change in APD_50_ (2935—75% prolongation) at low pacing frequencies (1–4 Hz). Though, at greater pacing frequencies (6–10 Hz) reductions in I_Ca_ lead to the greatest alterations in APD_50_ (62%–54% shortening), followed by reductions in I_Ks_ current (51%–34% prolongation) (Figure 13). Reductions in I_Kr_, during control and adrenergic stimulation conditions, yielded the lowest magnitude of APD_50_ change at all pacing frequencies (1% shortening to 10% prolongation). Equally, during 1–8 Hz and 1–4 Hz pacing under control and adrenergic stimulation conditions, respectively, reductions in I_Kr_ generated the lowest magnitude of APD_90_ change (3%–10% prolongation). During adrenergic stimulation, at higher pacing frequencies (6–10 Hz) however, I_to_ reductions had the lowest impact on AP late repolarization (5%–8% shortening) (Figure 14). During control conditions (1–10 Hz) and adrenergic stimulation (1–8 Hz), APD_90_ was influenced by the greatest magnitude (687—49% and 846—35% prolongation, respectively) through reductions in I_Ks_ (Figure 14). Figure 14 also shows that I_K1_ yields considerable influence (behind I_Ks_) on APD_90_ during control conditions at low frequencies (1–4 Hz) and adrenergic stimulation at almost all pacing frequencies (1–8 Hz), while I_Ca_ yields the second greatest magnitude of influence on APD_90_ at high pacing frequencies during control conditions and yields the greatest influence at the most strenuous pacing frequency during adrenergic stimulation.

Overall, the results displayed a tendency, in all ion channels (except I_Kr_), to yield a reduced impact on APD_50‐90_ with current block as pacing increased during control and adrenergic stimulation conditions. Meanwhile, the magnitude of APD_50‐90_ change caused by current block shows a relatively mixed pattern during adrenergic stimulation compared with control conditions depending on the ion channel manipulated.

**FIGURE 15:**
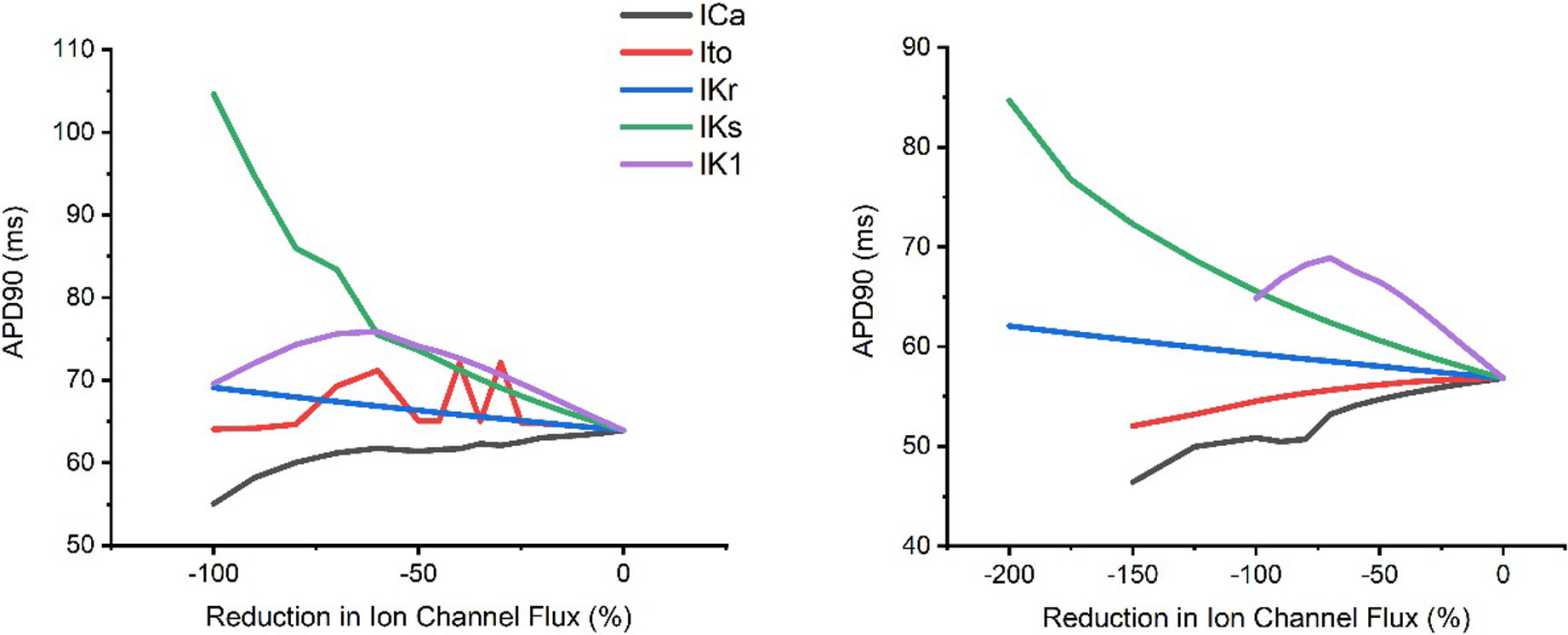
Modulations in APD_90_ under control (left) and adrenergic stimulation conditions (right) triggered by separate progressive ion channel flux block at 6 Hz pacing during sensitivity analysis modeling.

**FIGURE 16:**
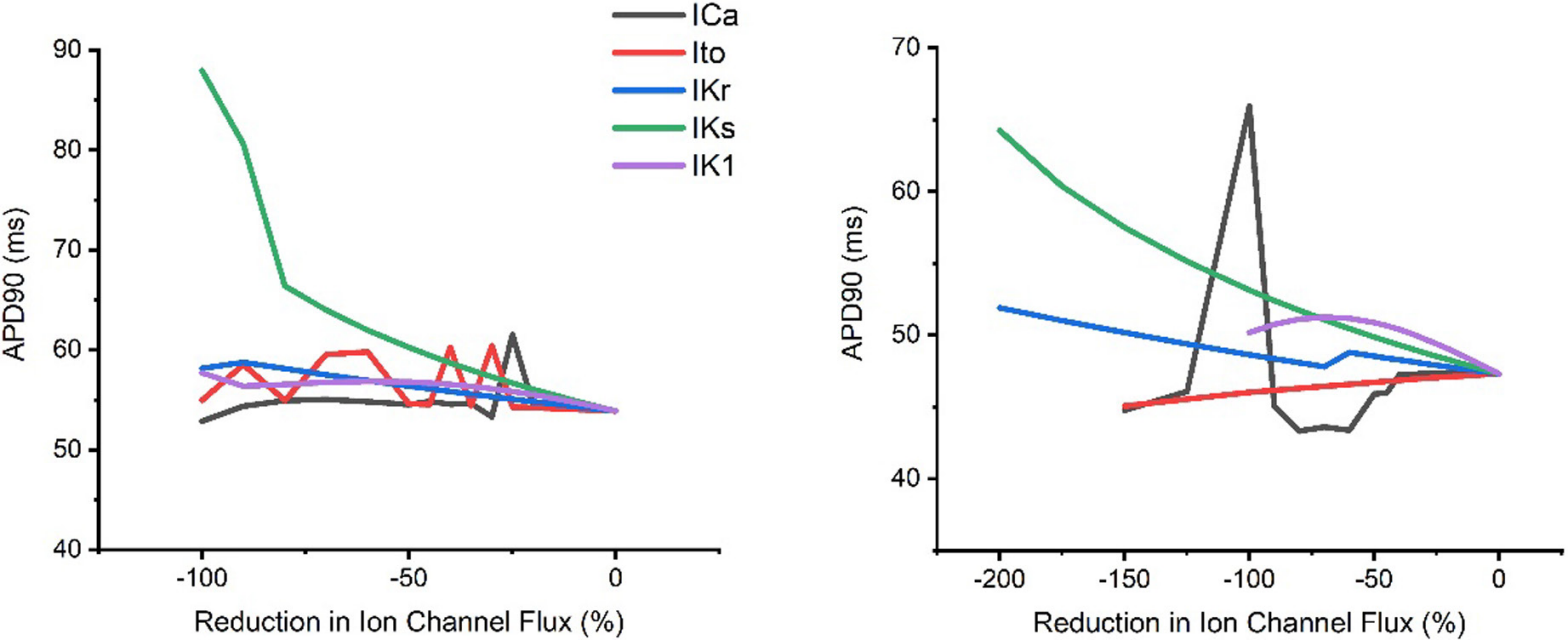
Modulations in APD_90_ under control (left) and adrenergic stimulation conditions (right) triggered by separate progressive ion channel flux block at 10 Hz pacing during sensitivity analysis modeling.

Figures 15 and 16 display the changes in late AP repolarization in response to ion channel block during control and adrenergic stimulation conditions at 6 and 10 Hz pacing which approximately emulates basal and strenuous exercising rat heart rates, respectively.

Figure 15 demonstrates the significant prolongation of late AP repolarization stimulated by I_Ks_ and I_K1_ block and the shortening of late AP repolarization stimulated by I_Ca_ block at 6 Hz pacing during control and adrenergic stimulation conditions. I_Kr_ block stimulated a steady gradual prolongation in late AP repolarization during control and adrenergic stimulation conditions at 6 Hz pacing. In contrast, I_to_ block demonstrated a steady shortening effect on late AP repolarization during adrenergic stimulation at 6 Hz pacing. However, during control conditions, a mixed initial impact on late AP repolarization was observed with I_to_ block at 6 Hz pacing, which eventualized as a slight shortening effect.

Figure 16 demonstrates the significant prolongation of late AP repolarization stimulated by I_Ks_ block at 10 Hz pacing during both control and adrenergic stimulation conditions. I_Kr_ and I_K1_ block showed a gradual prolongation effect on late AP repolarization at 10 Hz during both control and adrenergic stimulation conditions. I_to_ block during adrenergic stimulation at 10 Hz pacing stimulated a shortening effect on late AP repolarization but displayed a mixed impact during control conditions. Similarly, I_Ca_ block demonstrated a mixed effect on late AP repolarization at 10 Hz pacing during control and adrenergic stimulation conditions.

## 3 DISCUSSION

The aim of this study was to identify, in the adult rat, the ion channels that display the greatest response to variations in pacing and adrenergic stimulation and in turn identify the most dominant channels during such conditions, using a computational model of rat electrophysiology that can be paced at physiologically appropriate rates. This study also aimed to identify and quantify the influence of key ion channels involved in APD_50_ and APD_90_ responses to physiological stress.

The main findings of this work were multi‐faceted. Firstly, we have shown the mLR model is capable of more accurately modeling APD_50‐90_ at 1 Hz through to 10 Hz pacing during control and adrenergic stimulation conditions as well as the relative response to adrenergic stimulation (Howlett et al., 2022) compared with the original LR model. Secondly, we have shown the mLR model is more capable than the original LR model of simulating diastolic membrane potential that is comparable to experimental data during varied pacing and adrenergic stimulation, though further mLR model adjustment is required to accurately simulate AP amplitude that more closely aligns with the experimental data. Thirdly, we have shown the mLR model is better able to simulate similar peak I_Ca_ and I_K1_ currents to laboratory findings at 1 Hz pacing compared with the original LR model. Further, we have shown that I_to_ and I_Ca_ simulated by the mLR model are most dominant during control and adrenergic stimulation conditions. These currents also displayed the greatest responses to adrenergic stimulation followed closely by I_Ks_ in simulations by the mLR model. Meanwhile I_Ks_ and I_Kr_ simulated by the mLR model showed the greatest rate‐dependent responses during control and adrenergic stimulation conditions.

Also, we have shown in the mLR model, that during control and adrenergic stimulation conditions, AP plateau was predominantly most sensitive to changes in I_to_ and I_Ca_. In addition, AP late repolarization was most sensitive to changes in I_K1_ and I_Ca_ at low pacing frequencies and I_K1_ and I_Ks_ at higher pacing frequencies during control conditions. During adrenergic stimulation, AP late repolarization was most sensitive to changes in I_K1_ and I_Ks_. Moreover, AP plateau and late repolarization were least sensitive to changes in I_Kr_. Overall, APs were predominantly less sensitive to changes in ion current flux at higher pacing frequencies.

Finally, in the mLR model, we have shown I_Ks_ current block predominantly has the greatest magnitude of effect on AP plateau and late repolarization across all conditions and pacing, though I_Ca_ block yields superior influence on AP plateau at higher pacing frequencies during adrenergic stimulation. Total I_Kr_ block produces predominantly the smallest magnitude of effect on APs, though I_to_ block generates the smallest magnitude of effect on AP late repolarization during adrenergic stimulation at higher pacing frequencies. Once again, as pacing frequency increased, AP plateau and late repolarization were more resistant to modulations caused by ion current block.

### 3.1 Accurately simulating the rat ventricular action potential at a range of physiological pacing frequencies and during adrenergic stimulation

Figures 1 and 2 demonstrate the mLR model's ability to simulate the rat ventricular AP and response to changes in pacing frequency and superfusion of isoproterenol, providing the closest replication of experimental data from our laboratory trials (Howlett et al., 2022) at pacing frequencies equivalent to physiologically relevant rat heart rate ranges (~4–10 Hz). Figures 2 and 3 show the improved capability of the mLR model to simulate late AP repolarization during varied pacing and adrenergic stimulation that more closely resemble the experimental data compared with the original LR model. However, upon closer comparison, despite the relatively close resemblance of APD_90_ values simulated by the mLR model compared with the experimental data, differences in late AP repolarization response to increases in rate were observed. A gradual step‐wise decline in APD_90_ simulated by the mLR model can be observed in Figure 2, while experimental data display an initial prolongation in APD_90_ as pacing is increased from 1 Hz to 2 Hz during control conditions and 4 Hz during adrenergic stimulation conditions.

Figure 1 demonstrates the mixed ability of both the original LR model and the mLR model to simulate the AP plateau changes in response to varied pacing frequency and adrenergic stimulation. The mLR model was observed to more closely simulate APD_50_ within an acceptable range of experimental data at pacing frequencies that are reflective of basal physiological rat heart rates and lower. Meanwhile, the original LR model was observed to more closely simulate APD_50_ within an acceptable range of experimental data at the highest pacing frequencies which are reflective of physiological rat heart rates during strenuous exercise. Though it is important to recognize the similarity of APD_50_ rate‐dependency simulated by the mLR model and the contrast in APD_50_ rate‐dependency simulated by the original LR model compared with the rate‐dependency demonstrated by the experimental data, the ability of the original LR model to simulate APD_50_ at higher frequencies within the 95% confidence interval of experimental data compared with the mLR model highlights an important area of further necessary model development. Further modification in this regard is particularly important, given the steps forward taken to develop an electrophysiological rat ventricular myocyte model that simulates APD at pacing frequencies more reflective of physiological rat heart rates during exercise which provides (to some extent) progression from currently available rat heart models.

Though the ability of the mLR model to simulate APD, particularly late AP repolarization, at a range of pacing frequencies during control conditions and adrenergic stimulation that more closely resemble the experimental data compared with the original LR model, such changes highlight the need for further model adaptation. Further work is primarily required to more effectively map changes in AP plateau in response to varied stress, with further adjustments also required to more directly emulate the rate‐dependency of late AP repolarization exhibited in laboratory data.

When comparing mLR simulated APD data to existing literature, the APD_50‐90_ values simulated by the mLR model at low pacing frequencies (<4 Hz) are supported by a number of previous studies (APD_50_: 21–26 ms, APD_90_: 39.7–76 ms) (Şengül Ayan et al., 2020; Shigematsu et al., 1997; Wang & Fitts, 2017). Some existing literature also supports APD_90_ values simulated by the mLR model at higher (>4 Hz) pacing frequencies (~40–76 ms) (Shigematsu et al., 1997; Wang & Fitts, 2017), with studies also providing support for the rate‐dependent alterations of AP plateau (Fauconnier et al., 2003; Shigematsu et al., 1997) and late repolarization as pacing is elevated (Hardy et al., 2018; Wang & Fitts, 2017). However, there have been few rat myocyte AP investigations at pacing frequencies above 1 Hz; therefore, further experimental investigation would be beneficial to further develop the computational model and increase its robustness. Further studies would also benefit the adrenergic response in AP values in the same way, though one relevant previous study reported contrasting findings to the mLR model in regard to the response in AP late repolarization at a low pacing frequency (1 Hz) triggered by adrenergic stimulation (Wang & Fitts, 2017). This study indicates a greater response is elicited in response to adrenergic stimulation (40% APD_90_ shortening; 10 nM isoproterenol) than simulated by the mLR model (13% APD_90_ shortening) though this disparity may be explained by differences in isoproterenol dose as the mLR model is based in part, on experimental findings in our laboratory using a 5 nM isoproterenol dose (Wang & Fitts, 2017). Yet, another study supports the magnitude of isoproterenol‐induced response in AP late repolarization simulated by the mLR model (8% shortening at 5 Hz vs. 14%–11% simulated by the mLR model at 4 and 6 Hz, respectively) (Kamada et al., 2019).

Figures 4 and 5 display clearly the differences between the ability of the original LR model and the mLR model to simulate AP amplitude and diastolic membrane potential. In particular, Figure 5 shows the mLR model was able to simulate to a considerably higher degree than the original LR model, diastolic membrane potential comparable to experimental data, particularly during control conditions, with simulated diastolic membrane potential falling outside of the 95% confidence interval of experimental data at 8 Hz pacing. The mLR model was also able to simulate diastolic membrane potential within an acceptable range of experimental data at 1–6 Hz pacing during adrenergic stimulation. While the usefulness of a rat heart model able to map key electrophysiological responses to pacing at rates emulating physiological basal heart rates is certainly not of trivial benefit, the progression of robust models in the rat must be focused on the ability to fully map all AP variables and their responses to physiologically relevant stress/emulated exercise, highlighting a need for further adjustment of the mLR model. Moreover, while the modifications applied to the original LR model during the development of the mLR model helped to reduce the magnitude of AP amplitude and better resemble the AP amplitude responses to alterations in pacing (particularly during control conditions) and adrenergic stimulation in general, mLR simulated AP amplitude is still some‐way from accurately emulating AP amplitude modulations observed in experimental data during varied physiologically relevant stress and similarly highlights the need for further model development.

Existing Literature appears to agree with AP amplitude data simulated by the mLR model and AP amplitude data from laboratory investigations (91.6 mV–117.9 mV), with previous research also suggesting a reduction in AP amplitude with increases in pacing frequency and providing relatively close comparison to the values outlined in this study (Bouchard et al., 2004; Fauconnier et al., 2003; Jourdon & Feuvray, 1993; Liu et al., 2000; Ravens and Wettwer, 1998; Walker et al., 1993; Watanabe et al., 1983; Xu et al., 2016). However, it is important to note that the majority of existing data is based on AP amplitude data recorded at low pacing frequencies.

Existing literature appears slightly more mixed regarding its similarity to the diastolic membrane potential findings laid out in this work. Some research indeed points toward similar magnitudes of diastolic membrane potential as provided by the experimental data and mLR model simulations in this study (−62.9 mV to −71.6 mV) (0.1–0.5 Hz) (Jourdon & Feuvray, 1993; Liu et al., 2000; Walker et al., 1993). However, some other studies have demonstrated more negative diastolic membrane potentials, such as those simulated by the original LR model (0.2–1 Hz) (−80 to −80.4 mV) (Bouchard et al., 2004; Xu et al., 2016; Watanabe et al., 1983). However, once again, a great deal of existing data has been recorded at low pacing frequencies. Yet one study utilizing pacing frequencies toward more physiologically relevant rates (1–6 Hz) displayed diastolic membrane potential became less negative as pacing increased (measured −78 mV, −76 mV, −73 mV, and −71 mV at 1, 2, 4, and 6 Hz pacing, respectively; reflecting 3%, 6%, and 9% changes at 2, 4, and 6 Hz compared with 1 Hz pacing, respectively) (Fauconnier et al., 2003).

### 3.2 Using the mLR model to predict changes in ion currents with increases in pacing and superfusion of isoproterenol

Figures 11 and 12 demonstrate the varied projected peak current changes in I_Ca_, I_to_, I_Kr_, I_Ks,_ and I_K1_ as pacing frequency is elevated beyond traditionally investigated 1 Hz pacing as well as with the addition of isoproterenol. In Figures 11 and 12, alongside the individual current traces laid out in Figures 6, 7, 8, 9, 10, the greater ability of the mLR model to simulate ion current flux within relatively close margins of the laboratory data compared with the original LR model can be observed at low pacing frequencies. However, while this demonstrates some progression in mapping ion current responses using a computational model, these figures (Figures 6, 7, 8, 9, 10, 11, 12) also clearly highlight the need for further model development. Firstly, though improvements have been made in the ability of the mLR model to produce some ion currents (I_Ca_, I_K1_, I_Kr_) within a comparable magnitude to experimental data, the magnitude of other ion currents (I_Ks_) is not so closely emulated. Secondly, like studies exploring changes in APD, investigations in ion channel function have been predominantly limited to pacing frequencies at 1 Hz and below, and therefore, the mLR model would also greatly benefit from further experimental study to support peak current data simulated at more physiologically relevant pacing frequencies.

Available existing literature has often reported greater I_Ca_ magnitudes (>6 pA/pF) than those simulated by the mLR model at pacing frequencies up to 2.5 Hz (Jourdon & Feuvray, 1993; Liu et al., 2000; Meszaros et al., 1997; Sakatani et al., 2006; Scamps et al., 1990; Vizgirda et al., 2002; Walker et al., 1993; Xiao et al., 1994; Zhou et al., 1998). Yet, previous studies provide support to the relative isoproterenol‐induced response in peak I_Ca_ current simulated by the mLR model (mLR model: 67% vs. literature: 28%–120%) (Katsube et al., 1996; Sakatani et al., 2006; Scamps et al., 1990; Şengül Ayan et al., 2020; Vizgirda et al., 2002; Xiao et al., 1994; Zhou et al., 1998). Studies investigating I_to_ in rats have been far more frequent compared with investigations in most other K^+^ currents, though I_to_ investigations exploring adrenergic responses are few. The modulation of I_to_ by β‐adrenergic signaling is not completely clear, with some research suggesting I_to_ reduces in other mammals (Gallego et al., 2014; van der Heyden et al., 2006; Zhengrong et al., 2011). Though in canines, the I_to_ response to adrenergic stimulation has been suggested to depend on the specific component, with fast component amplitude reducing in magnitude while slow component amplitude increases in magnitude (Nakayama & Fozzard, 1988). Previous research has reported, at low pacing frequencies (0.1–0.5 Hz), peak I_to_ of 8.03 ± 3.75 pA/pF–31.0 ± 2.1 pA/pF in rats (Bénitah et al., 2001; Cho et al., 2017; Junior et al., 2017; Kilborn & Fedida, 1990; Tomita et al., 1994; Wettwer et al., 1993; Yokoshiki et al., 1997), providing some support to the values simulated by the mLR model at low pacing frequencies (1 Hz). Furthermore, limited research suggests the existence of a rate‐dependent reduction in I_to_ (Josephson et al., 1984; Shigematsu et al., 1997) between 0.2 and 5 Hz pacing (Shigematsu et al., 1997), further supporting the simulated findings of the mLR model. However, the findings of this study were based on I_to_ block on APD in current‐clamp mode and therefore do not provide current magnitudes (Shigematsu et al., 1997). The magnitude of rate‐dependent I_to_ reduction and changes occurring at greater pacing frequencies are unknown. Very little work on I_K1_ in rats has been carried out at even the lowest pacing frequency utilized in this study (1 Hz). Data from existing literature at frequencies <1 Hz have suggested peak outward I_K1_ current ranges considerably from approximately 0.5–6.5 pA/pF (Bébarová et al., 2014; Fauconnier et al., 2005; He et al., 2008; Jourdon & Feuvray, 1993; Kilborn & Fedida, 1990; Liu et al., 2000; Wahler, 1992), indicating simulated I_K1_ by the mLR model is situated at the low end of such a spectrum. Similarly, investigations at pacing frequencies <1 Hz have yielded peak I_Kr_ current magnitudes of 0.18–0.48 pA/pF (Sakatani et al., 2006; Wang & Fitts, 2020), indicating I_Kr_ values simulated by the mLR model once again most closely relate to lesser magnitudes of previous investigations (Wang & Fitts, 2020). However, the limited findings demonstrating I_Kr_ response to adrenergic stimulation (100 nM isoproterenol) in the rat provide much closer comparison to the adrenergic response simulated by the mLR model at low pacing (17%–45% vs. 35%) (Sakatani et al., 2006; Şengül Ayan et al., 2020). Existing I_Ks_ findings correlate relatively well with mLR model simulations in the rat, though there exists some indication that simulated I_Ks_ is slightly underestimated, as research has suggested peak I_Ks_ magnitudes of 0.71–1 pA/pF (Sakatani et al., 2006; Wang & Fitts, 2020), though once more these findings from existing literature are based on low frequency voltage clamp trials, which of course may underlie some deviations. Equally, previous research has reported greater relative responses in I_Ks_ to adrenergic stimulation (5 nM isoproterenol) (50%–54%) compared with the relative response simulated by the mLR model (35%) at low frequencies (Wang & Fitts, 2020), though the results of another study provide closer comparison (38% increase in peak I_Ks_) to the adrenergic response simulated by the mLR model (Sakatani et al., 2006).

### 3.3 Ion currents underlying AP plateau responses to increased pacing and adrenergic stimulation

The results of the mLR model sensitivity analysis suggest I_to_ and I_Ca_ require the lowest magnitude of block before triggering at least a 5% change in APD_50_ at most pacing frequencies under control and adrenergic stimulation conditions (Tables 1 and 2). These findings are indicative of key roles in the modulation of AP plateau in response to elevations in pacing and isoproterenol superfusion as would occur normally in response to exercise. Such findings were expected given that both currents are widely regarded to operate maximally during early repolarization and the plateau phases of the AP (Bers, 2002; Grant, 2009). These findings also corroborate with the individual magnitude of flux changes mentioned previously.

Similarly, the increasing involvement of I_Ks_ during physiological stress on AP plateau, particularly during situations emulating high intensity exercise (increased pacing + adrenergic stimulation), was expected given that previous recent research has supported the dominance of I_Ks_ in AP repolarization during adrenergic stimulation (Banyasz et al., 2014; Wang & Fitts, 2020) and was evidenced in this study by the increasing sensitivity of the AP plateau to I_Ks_ flux block. The role of I_Ks_ in AP plateau was further highlighted by the findings in this study suggesting I_Ks_ block leads to the greatest magnitude of APD_50_ change of all currents investigated at most pacing frequencies under control and adrenergic stimulation conditions. Typically, I_Ca_ block provided similar and at times even greater magnitudes of APD_50_ change. The sensitivity of I_Ks_ is unsurprising given the increased current observed in this study with increments in pacing and the introduction of isoproterenol, particularly during pacing beyond the typical resting rat heart rate (6 Hz), where peak current increased exponentially alongside the additive current increase induced by isoproterenol stimulation, stimulating a 75%–113% current increase in response to mimicked exercise (6 vs. 8–10 Hz + adrenergic stimulation).

The sensitivity of AP plateau phase to changes in I_to_ and I_Ca_ likely explains the slight prolongation in simulated APD_50_ at pacing frequencies of 4 Hz and below, as I_Ca_ increases mildly while I_to_ consistently reduces in current magnitude, potentially allowing a net prolongation in APD (Figures 1 and 3). Meanwhile, the shortening of APD_50_ at pacing frequencies reflecting physiological rat heart rate ranges (with and without isoproterenol superfusion) is likely a result of the moderate increments in I_Ks_ compared with the gradual falling of I_Ca_ (Figures 1 and 3).

An interesting finding was that the AP plateau was least sensitive to I_Kr_ and that I_Kr_ block also provided the lowest magnitude of effect on APD_50_, often yielding no effect at all (Tables 1 and 2). This was despite similar current responses to I_Ks_ during pacing and adrenergic stimulation emulating physiological exercise (6 vs. 8–10 Hz + adrenergic stimulation) and contrary to a much more exaggerated current response to elevations in pacing even from the lowest of frequencies (Figure 3). Such results indicate the dominance of I_Ks_ as a key current responsible for modulating repolarization in times of physiological stress followed by I_K1_ and finally I_Kr_ and also indicates a minimal role for I_Kr_ in mediating the Ca^2+^: K^+^ balance and in turn phase 2 (and maybe phase 1) of the AP.

### 3.4 Ion currents underlying AP late repolarization responses to increased pacing and adrenergic stimulation

The results of the mLR model sensitivity analysis suggest I_K1_ and I_Ca_ require the least magnitude of block to stimulate a 5% alteration in AP late repolarization at pacing frequencies lower than resting rat heart rates (<6 Hz) during control conditions (Tables 1 and 2). At pacing frequencies greater than resting rat heart rates to pacing reflecting physiological exercise performance (>6 Hz + Iso), APD_90_ is most sensitive to modulations in I_K1_ and I_Ks_ and likely underlies the considerable shortening (coupled with the gradual decline of APD_90_ sensitivity to I_Ca_ and gradual I_Ca_ current decline after 6 Hz) that occurs as incremental stress (increasing pacing and superfusion of isoproterenol) is applied to the model myocyte (Figures 2, 14, 15, 16).

Similarly, I_Ks_, I_K1,_ and I_Ca_ yield the greatest impact on AP late repolarization during current block, highlighting the significant role of this trio of currents on structuring APD and its response during episodes of physiological stress that mimic exercise performance. During simulations emulating resting rat heart rates, I_Ks_ and I_K1_ yield the greatest impact on late repolarization. Though, during simulations emulating, in part, physiological rat exercise (>6 Hz pacing) the impact of I_Ks_ on APD_90_ is followed by I_Ca_, however when increased pacing is combined with isoproterenol superfusion, as would occur during adrenergic stimulation in vivo, I_K1_ yields greater impact (second to I_Ks_) than I_Ca_ until maximal pacing is applied (Figures 14, 15, 16). This displays the dominance of repolarizing K^+^ channels in shaping APD during physiological conditions emulating exercise as would be in vivo. As with the AP plateau phase, the sensitivity of I_Ks_ corroborates the individual rate‐dependent and adrenergic stimulation‐induced current responses (Figures 2, 11 and 12) and subsequently generates considerable influence on late repolarization during myocyte simulations above resting rat heart rates. The consistent mild changes in I_K1_ current with increments in rate and the introduction of adrenergic stimulation highlight the level of sensitivity and in turn, responsibility this current has in helping to control late repolarization and subsequent APD (Figures 11 and 12).

Once again, the findings of this study highlight the lack of effect alterations in I_Kr_ have on late repolarization, despite exponential current increases which greatly overshadow the relative modulations observed in all other currents investigated (Figure 3). Again, this could be indicative of a minimal role for I_Kr_ in determining the outcome of the inward and outward current balance during late‐stage repolarization and subsequent determination of APD.

Overall, the results of this study suggest I_to_ and I_Ca_ are responsible for the greatest input regarding the control of the AP plateau, and I_K1_ and I_Ca_ have significant roles in determining the shape of late AP repolarization. Meanwhile, I_Ks_ appears to have a considerable role in the modulation of AP plateau and a major/primary role in the modulation of late AP repolarization, particularly during conditions reflective of physiologically relevant exercise in the rat (>6 Hz + adrenergic stimulation) (Figures 15 and 16). The individual changes in these currents in response to increments in pacing and the addition of adrenergic stimulation underlie the changes observed in simulated APD_50_ and APD_90_ shown in Figures 1 and 2. The shortening of APD_50_ and APD_90_ at pacing frequencies equivalent to resting rat heart rates and above likely demonstrates the shift in Ca^2+^: K^+^ current balance, whereby repolarizing K^+^ channels tend to dominate control over APD.

## 4 PRACTICAL APPLICATIONS

Generating a robust computational model of rat ventricular myocyte electrophysiology is vital to most effectively continue to improve knowledge and understanding in the field of cardiac electrophysiology. Given that the rat is a very commonly used animal model for studying cardiac physiology and pathophysiology (Flister et al., 2015; Huang et al., 2011; Sallé et al., 2008), the more information gathered, the better our understanding of what initiates arrhythmias, disease and other conditions and also specifically how they limit function. While further study is required to continually improve model validity and reliability, the mLR model is able to provide important physiologically relevant insight on the responses to computational emulations of physical activity and exercise. Such a model opens up the possibility of investigating multiple electrophysiology variables at a single time, and the subsequent vast individual responses to physiological stress as would be encountered potentially multiple times a day in daily life. While the eventual goal in this field is to develop similar models based on humans in order to reduce animal use in scientific research, it is imperative that robust models based on one of the most used animal models is developed and available as a priority, as arguably, this will provide initial large reductions in the need for animals in basic science research, through the associated experimental streamlining of early research. In addition, this study with added future work may enable development of whole‐heart models. The work in this study may also benefit the study of cardiac dysfunction and disease due to the impairment of exercise response known in heart failure and aging as well as the impairment of repolarizing K^+^ channels and subsequent repolarization in a number of arrhythmias (Chiamvimonvat et al., 2017; Cho et al., 2017; Farrell & Howlett, 2008; Ferrara et al., 2014; Howlett & Lancaster, 2021; Jeevaratnam et al., 2017; Najafi et al., 2016; Steenman & Lande, 2017). Lastly, findings from the sensitivity analysis may help direct future study of the key ion channels influencing AP modulation highlighted in this study and provide information on areas of therapeutic interest.

## 5 LIMITATIONS

Due to the lack of AP investigations documenting APD in response to physiological doses of adrenergic stimuli, particularly during pacing at physiological activation frequencies relevant to the test‐species, under physiologically relevant environmental conditions, the mLR model was developed, in part, using data from our laboratory experiments (Howlett, 2022; Howlett et al., 2022). Typically, novel computational models would be validated against a range of relevant data from various studies; therefore, future studies would help further, more robustly, develop and validate the mLR model.

Equally, the study of ion channels is typically performed during voltage‐clamp experiments primarily at pacing frequencies under 1 Hz (though sometimes at 1 Hz), which means the majority of evidence in existing literature has been acquired at pacing frequencies incredibly far away from those that would be relevant to the most common test‐species (rats and mice). This creates difficulty in providing adequate comparison in the literature to simulated ion currents from modeling experiments and makes the validation of important model components almost impossible, without using estimations or cross species information. In addition, such a lack of vital information also creates difficulty in initial model generation. The recent development of the “so‐called” onion peeling variation of the AP clamp technique, also known as the AP clamp sequential dissection technique (Banyasz et al., 2011, 2014; Chen‐Izu et al., 2012), whereby multiple ion channels can be investigated by sequentially blocking channels of interest while being stimulated by the myocyte's own AP may lead to further more physiologically relevant future studies at pacing frequencies that more closely reflect the heart rate ranges of donor‐hearts.

In order to provide the required myocyte stability for AP recordings at such a range of pacing frequencies (1–10 Hz), the pipette solution included 10 mM EGTA. Such a dose of EGTA, while not at all unusual in whole‐cell patch‐clamp electrophysiological experiments, interferes with the intracellular milieu and may influence AP repolarization and harm physiological relevance in the wider context. This potential issue has been outlined in one study which indeed provided similar rate‐dependent APD changes (0.2–5 Hz pacing) during whole‐cell patch‐clamp recordings (using 11 mM EGTA) on rat ventricular myocytes to those demonstrated by the experimental and simulated data presented in this work; however, the rate‐dependent APD prolongation that occurred throughout the lower pacing frequencies was seemingly ameliorated when recordings were repeated on papillary muscle using sharp electrophysiological techniques (Shigematsu et al., 1997). This suggests similar APD values to those reported in this study throughout a range of pacing frequencies may not be reproduced without intracellular Ca^2+^ chelation as may occur in vivo (Shigematsu et al., 1997) and should therefore be considered when translating the outcomes of the mLR model simulations to the wider context of electrophysiological exercise responses as the experimental data (Howlett et al., 2022), in part, facilitated mLR model development. Although future studies would indeed benefit from performing similar experiments using techniques that influence the true intracellular environment to a lesser extent and in turn adapting the mLR model, it should be understood that myocyte isolation techniques as well as any disturbance to membrane integrity or intracellular/extracellular environment is likely, to some extent, to impact key components such as intracellular Ca^2+^ and in turn the overall physiological relevance of subsequent recordings due to the inherent removal of buffering and electrotonic support from other cells. Furthermore, the mLR model in its current state is capable of providing potentially vital insight to the rat ventricular myocyte response at higher pacing frequencies and may help, to some extent, the mapping of the key electrophysiological component responses to (emulated) strenuous exercise in a widely used animal model in cardiovascular research.

As outlined in the methodology (Section 7), the mLR model was developed, in part, using laboratory data from our previous experiments, from which I_Ks_, I_Kr,_ and I_K1_ currents have been assumed from chromanol 293b‐sensitive, E4031‐sensitive and BaCl_2_‐sensitive current recordings from AP‐clamp investigations, respectively (Howlett, 2022). While such blockers have been widely used and indeed effectively block these K^+^ channel currents, further voltage‐clamp experiments using more advanced/specific blocking techniques may be required to reliably confirm the absence of current contamination. For example, some research indicates chromanol 293b‐sensitive current at the dose used (30 μM) may be attributed somewhat to I_Kur_ and I_to_ current as well as I_Ks_ current (Árpádffy‐Lovas et al., 2022; Bosch et al., 1998; Du et al., 2003; Sun et al., 2001). This may impair the ease of interpretation of individual repolarizing K^+^ channel current findings from mLR model simulations, though the mLR model still allows potentially valuable information to be obtained regarding AP repolarization and overall K^+^ current modulations in response to emulated exercise.

Furthermore, the incorporation of I_Kr_ and I_Ks_ coupled with the omission of other repolarizing K^+^ channels that are vital to rat ventricular myocyte AP modulation such as I_Kur_ and I_ss_ should also be considered (Árpádffy‐Lovas et al., 2022; Choisy et al., 2004; Himmel et al., 1999; Sakatani et al., 2006). I_Ks_ and I_Kr_ were incorporated into the model as recent research has pointed toward the potential greater relevance of rectifying K^+^ channels (particularly I_Ks_) and their modulation of AP repolarization in rat ventricular myocytes, particularly during adrenergic stress (Olgar et al., 2022; Wang & Fitts, 2020), despite earlier studies suggesting a minimal role for such ion channels in rats (Joukar, 2021; Pond et al., 2000; Regan et al., 2005; Tande et al., 1990; Varró et al., 1993). However, I_Ks_ and I_Kr_ were incorporated into the model at the expense of I_ss_ as a practical method to investigate channels of interest and avoid de‐stabilizing the balance of K^+^ currents established by the original LR model (Stevenson‐Cocks, 2019). Given the understood independence of I_ss_ from other repolarizing K^+^ channels, its replacement may negatively influence the accuracy of mLR simulations (Choisy et al., 2004; Himmel et al., 1999; Sakatani et al., 2006). Further research combined with further model development including the incorporation of the full range of ion channels expressed in the rat ventricle will benefit the model accuracy and improve physiological relevance and allow important information from mLR model simulations to be extracted and enable the mapping of electrophysiological responses to exercise in rats that better represents true physiological responses and improves translational value of model outcomes to the wider context.

While it is extremely valuable to develop electrophysiological models of the rat heart due to the wide use of the rat as a model, the translation of outcomes to human physiology is vital. However, the known differences in AP waveform and overall APD between rats and humans impair the ease of translation (Árpádffy‐Lovas et al., 2022; Varró et al., 1993). Such differences in AP waveform are a result of varied ion channel expression alongside the varied influence of certain ion channels. For example, I_to_ expression as well as I_to_, I_K1,_ and I_Kur_ influence on AP repolarization is greater in rats compared with humans (Árpádffy‐Lovas et al., 2022; Varró et al., 1993). Similarly, I_Kr_ is responsible (to a considerable degree) for AP repolarization in humans, while I_Kr_ likely has a minimal role in rat AP repolarization, and its measurement in rats has been suggested to be difficult (Árpádffy‐Lovas et al., 2022; Howlett, 2022; Jost et al., 2013; Joukar, 2021; Wang & Fitts, 2020). Such differences should be thoroughly considered when interpreting and translating mLR model simulations to the wider context.

## 6 CONCLUSION

The mLR model provides relatively accurate simulations of the rat ventricular myocyte AP under control and physiologically relevant conditions of adrenergic stimulation (with combined superfusion of isoproterenol and increments in pacing up to 10 Hz) comparable (at least qualitatively) to experimental results achieved in patch‐clamp, based on ion channel dynamics embedded in existing literature and experimental findings from our laboratory, reflecting environmental conditions that closely mimic the experimental patch‐clamp set‐up. The results of further analysis demonstrate the importance of I_to_ and I_Ca_ as well as I_K1_ and I_Ca_ in controlling the plateau and late repolarization phases of the AP, with I_Ks_ yielding a major influence on both phases, particularly during simulations emulating adrenergic stimulation (increments in pacing and isoproterenol superfusion) as would be observed in vivo during exercise. This work will assist in the study of arrhythmia and disease and may function as a beneficial predictive tool in identifying areas of therapeutic interest. Future acquisition of ion channel data during experimental study at more physiologically relevant pacing frequencies and isoproterenol doses along with the incorporation of the full ensemble of ion channels in the rat heart will help to further strengthen this model and enable the generation of key reference values for AP variables, vital for dysfunction and disease research.

## 7 METHODS

### 7.1 Experimental methods

#### 7.1.1 Experimental animals

Experiments were performed on male Wistar rats (3 months of age; 146–215 g). Rats were housed in the Animal Unit, Faculty of Biological Sciences, University of Leeds. Food and water were provided ad‐libitum, and rats were subject to 12‐h light: dark cycles. Rats were sacrificed by concussion of the brain and cervical dislocation in accordance with Schedule One methods stated in the Animals (Scientific Procedures) Act, 1986 and approved by the ethics committee at the University of Leeds. All data presented as mean ± standard error of the mean (SEM). Confidence intervals were calculated using the formulae SEM × 1.96.

#### 7.1.2 Isolation of myocytes

Male Wistar rat ventricular myocytes were isolated following the methods laid out in our previous work (Howlett, 2022; Howlett et al., 2022). All materials, unless otherwise stated, were purchased from Sigma‐Aldrich. Solution molarity displayed as mM unless otherwise stated. All solutions perfused using Langendorff methods were bubbled with Oxygen (100%; BOC). Briefly, after sacrifice, rat hearts were placed in a Ca^2+^‐free Isolation Solution (NaCl 130, KCl 5.4, MgCl_2_ 1.4 (Fluka), NaH_2_PO_4_ 0.4 (Acros Organics), HEPES 10, Glucose 10, Taurine 20, Creatine 10, pH titrated to 7.3 using 1 M NaOH (BDH Laboratory Supplies)) before cannulation via the aorta and retrograde constant flow perfusion with perfusion solution (isolation solution +750 μM CaCl_2_) at 37°C using the langendorff method (3.5–4 mLs per minute) until blood was removed. Following this, hearts were perfused (3 min) with Ca^2+^ buffer solution (Ca^2+^‐free isolation +100 μM EGTA) prior to perfusion (8–10 min) with enzyme‐containing solution (50 mL Ca^2+^‐free isolation solution, 50 mg BSA, 5 mg protease, 50 mg collagenase (type 2 Worthington)).

Ventricles were then removed, dissected, finely chopped, shaken at 37°C in enzyme‐containing solution for 5 min, filtered (nylon gauze 200 μM) and suspended in perfusion solution until use.

#### 7.1.3 Electrophysiology

The electrophysiological set‐up and subsequent protocols were the same as outlined in our previous work (Howlett, 2022; Howlett et al., 2022). Briefly, rat ventricular myocytes were superfused (3 mL per minute (Fujisawa et al., 2000)) with Tyrode solution (NaCl 136, KCl 4, MgCl_2_ 2, CaCl_2_ 1, HEPES 10, Glucose 10, pH titrated to 7.4 using 1 M NaOH) at 37°C under control conditions. Glass pipettes containing: KCl 135, EGTA 10, HEPES 10, glucose 5 (pH titrated to 7.2 using KOH (1 M) (Alfa Aesar)), attached to an AxoPatch 200B patch‐clamp amplifier (Axon CNS, Molecular Devices) stimulated ventricular myocytes. All electrophysiological data were digitized using pCLAMP 9.0 software (Digidata 1440A, Axon CNS, Molecular Devices). All data were obtained from ventricular myocytes using whole‐cell patch‐clamp techniques. Action potential and ion channel current data were sampled at 5000 KHz after low‐pass filtering at 1 KHz. Liquid junction potential was calculated (~7 mV) but not adjusted for. Low resistance pipettes (4–8 MΩ) were used for all experiments. Series resistance was monitored compensated for (≥80%) throughout (Armstrong & Gilly, 1992).

#### 7.1.4 Action potential recordings

Action potentials were evoked in current‐clamp by 1.5 nA (2 ms) current pulses. Action potentials recordings were made via repeated sweep sequences after 30–90 s preconditioning at every frequency. In every cell, 3–5 steady‐state AP recordings were achieved under control conditions at 1 Hz followed by 2, 4, 6, 8, and 10 Hz pacing frequencies. Isoproterenol (5 nM) was then introduced, and AP recordings were repeated in the same sequence. Isoproterenol was superfused at the same flow rate as Tyrode (3 mL/min) and 5 min were allowed after the introduction of isoproterenol before recordings were made to allow for drug equilibration (Harding et al., 1988; Johnson et al., 2012; Lim et al., 1999). Action potential recordings were averaged for each pacing frequency under control and adrenergic stimulation conditions and analyzed in Clampfit 10.7 software. Time taken for the AP to return to 50% and 90% of the diastolic voltage after depolarization (APD_50_, APD_90_) was the primary variables measured. Action potentials were successfully recorded in 19–22 cells (n) in eight rats (N). Average access resistance was 19 ± 1.3 MΩ. Average cell capacitance was 142 ± 9.6 pF.

#### 7.1.5 Ion current recordings

Peak I_Ca_ was measured in rat ventricular myocytes (*n* = 36; *N* = 6) during a voltage‐clamp protocol (1 Hz) consisting of 200 ms 10 mV square pulse‐steps from a holding potential of −50 mV after a pre‐pulse from −80 mV to −50 mV to remove Na^+^ current. After a 1–2 min preconditioning period, ~5 recordings (10 sweeps per recording sequence) were obtained during Tyrode superfusion (control) (3 mL/min). Isoproterenol (100 nM) was then superfused (3 mL/min) for 5 min before recordings were repeated (Harding et al., 1988; Johnson et al., 2012; Lim et al., 1999). Recordings during control and adrenergic stimulation conditions were averaged and analyzed offline using Clampfit software. All I_Ca_ data were normalized to cell capacitance (I/Cm). Average access resistance was 24 ± 2.1 MΩ. Average cell capacitance was 150 ± 7.5 pF.

Peak chromanol 293b‐sensitive, E4031‐sensitive, and BaCl_2_‐sensitive currents were measured in rat ventricular myocytes (*n* = 16, *N* = 8) using a sequential dissection (onion peeling) technique during AP‐clamp experiments (Banyasz et al., 2011, 2014). The cells own steady‐state APs during control and adrenergic stimulation conditions—recorded initially using a 2 ms 1.5 nA pulse after a 3‐min preconditioning period—were used to stimulate myocytes throughout current recordings (1 Hz). Chromanol 293b (30 μM), E4031 (1 μM), and BaCl_2_ (50 μM) were superfused (3 mL/min) sequentially (Banyasz et al., 2011; 2014), and the compensatory currents elicited in response were recorded. Two three minutes were allowed after each blocker was introduced to enable drug equilibration. After all control recordings were complete, drug washout was facilitated by Tyrode superfusion (3 min), before isoproterenol (100 nM) superfusion began. Five minutes separated the introduction of isoproterenol and the recording of the new steady‐state self‐AP stimulus, to allow for drug equilibration. Once the new steady‐state AP was set as the stimulus, blockers were superfused in the exact same way (with continued isoproterenol superfusion) and compensatory currents were recorded once more. Compensatory current recordings were subtracted from the recorded baseline current (under control and adrenergic stimulation conditions) as well as previous compensatory current recordings to identify peak chromanol 293b‐sensitive, E4031‐sensitive and BaCl_2_‐sensitive currents. All data were normalized to cell capacitance (I / Cm). Average access resistance was 27 ± 1.3 MΩ. Average cell capacitance was 142 ± 11.4 pF. Peak chromanol 293b‐sensitive, E4031‐sensitive, and BaCl_2_‐sensitive currents were assumed to reflect I_Ks_, I_Kr,_ and I_K1_ currents, respectively.

### 7.2 Computational methods

#### 7.2.1 Computational model structure

The mLR model utilized in this study was developed from the original LR model (Stevenson‐Cocks, 2019) using data from existing literature and data from our laboratory recently made available (Section 2.0) (Howlett, 2022). Details of the LR model are presented in Data S1; the mLR model, as C code, is available in Data S2. The original LR model was based on the work carried out by Colman et al. (2017), Gattoni et al. (2016) and enabled the simulation of the rat ventricular AP at pacing frequencies that reflect rat HRs during strenuous exercise (Stevenson‐Cocks, 2019). The mLR model was developed/modified in order to incorporate new AP data recorded at 10 Hz pacing alongside the new data demonstrating the response to adrenergic stimulation in AP at higher pacing frequencies in more recent literature (Howlett et al., 2022; Wang & Fitts, 2017). Further, more recently acquired ion channel data from our laboratory studies investigating responses to adrenergic signaling were also used to develop the mLR model (Howlett, 2022) (Section 2.0). Ultimately, this development was required to better align model simulations with experimental data, previously unavailable during development of the original LR model and also to better align the model simulation conditions with experimental data collection conditions used within our laboratory, derivatives of which are used elsewhere in the literature.

#### 7.2.2 Model modifications

The modifications applied to the original LR model (Stevenson‐Cocks, 2019) in order to develop the mLR model are described below. These modifications have also been outlined in a previous publication (Howlett, 2022). In order to reproduce experimental conditions (Howlett, 2022; Howlett et al., 2022), extracellular ionic concentrations were set to [Na^+^]_o_ = 136.0 mM, [Ca^2+^]_o_ = 1.0 mM, [K^+^]_o_ = 4.0 mM (LR model: [Na^+^]_o_ = 140.0 mM, [Ca^2+^]_o_ = 1.8 mM, [K^+^]_o_ = 5.4 mM). Using formulae from Korhonen et al. (2009), the steady‐state potassium (K^+^) current (I_ss_) in the LR model was split to provide the slow and rapid delayed rectifier K^+^ currents (I_Ks_ and I_Kr,_ respectively). In order to prevent the disturbance of repolarization dynamics, but also correlate with experimental ion current and APD data (Howlett, 2022; Howlett et al., 2022), the peak current (during steady‐state APs at 1 Hz pacing) balance of potassium currents was constrained to remain at I_to_: I_K1_: I_Kr_: I_Ks_ = 88.7%: 6.4%: 0.9%: 4.0%, that is, matching the peak current balance from the original LR model (I_to_: I_K1_: I_ss_ = 88.7%: 6.4%: 4.9%) (Stevenson‐Cocks, 2019) and the I_Ks_: I_Kr_ balance observed in experimental settings from work in our laboratory (Howlett, 2022) and in existing literature (I_Ks_~4–4.5 fold > I_Kr_) (Wang & Fitts, 2020). Potassium current maximal conductances for the mLR model are shown in Table 3. In order to reproduce experimental APD restitution dynamics (Howlett et al., 2022), the activation time constant of I_Ks_ was set to 100 ms. Model state variable initial conditions were obtained by allowing the mLR model to run unpaced (i.e., quiescent) until all state variables became stable. These initial conditions are given in Table 4.

**TABLE 3:** Maximal membrane current conductance ($\bar{g}$) in the mLR model and the LR model (Stevenson‐Cocks, 2019). Table re‐printed from Howlett (2022) and Stevenson‐Cocks (2019).

Ion channel	Modified LR model	Original LR model
$\bar{g}$_Na_	7.0 mS/μF	7.0 mS/μF
$\bar{g}$_to_	0.116 mS/μF	0.196 mS/μF
$\bar{g}$_ss_	N/A	0.12 mS/μF
$\bar{g}$_Kr_	0.13 mS/μF	N/A
$\bar{g}$_Ks_	0.295 mS/μF	N/A
$\bar{g}$_K1_	0.000017 S	0.00004 S
$\bar{g}$ Na^+^: K^+^ ATPase	13.8 mS/μF	13.8 mS/μF

**TABLE 4:** Initial conditions in the mLR model.

Parameter	Description	Initial state
V	Membrane potential (mV)	−67.843053
I_na_m_	I_Na_ activation gate m	0.028908
I_na_h_	I_Na_ inactivation gate h	0.204194
I_na_j_	I_Na_ inactivation gate j	0.204194
I_to_r_	I_to_ activation gate r	0.006610
I_to_s_	I_to_ inactivation gate s	0.963549
I_to_slow_	I_to_ inactivation gate slow	0.963549
I_f_y_	I_f_ inactivation gate y	0.001168
Ca__cyto_	Ca^2+^ concentration in cytoplasm (μM)	0.074005
Ca__ss_	Ca^2+^ concentration in subspace (μM)	0.074134
Ca__ds_	Ca^2+^ concentration in dyadic cleft (μM)	0.074844
Ca__jsr_	Ca^2+^ concentration in junctional SR (μM)	773.296210
Ca__nsr_	Ca^2+^ concentration in network SR (μM)	773.296210
LTCC__d1_	I_Ca_ activation state d1	0.999972
LTCC__d2_	I_Ca_ activation state d2	0.000027
LTCC__d3_	I_Ca_ activation state d3	0.000001
LTCC__f_	I_Ca_ voltage induced inactivation state f	0.999509
LTCC__fca_	I_Ca_ Ca^2+^ induced inactivation state fca	0.984678
RyR__CA_	RyR “closed” activated state	1.0
RyR__OA_	RyR “open” activated state	0.000004
RyR__OI_	RyR “open” inactivated state	0.0
RyR__CI_	RyR “closed” inactivated state	0.0
RyR__M_	Monomer binding (mM)	0.000047
RyR__Mi_	Monomer binding inactivation (mM)	0.000496
P0	Permissive tropomyosin with 0 cross bridges	0.001667
P1	Permissive tropomyosin with 1 cross bridge	0.001442
P2	Permissive tropomyosin with 2 cross bridges	0.002692
P3	Permissive tropomyosin with 3 cross bridges	0.002345
N0	Nonpermissive tropomyosin with 0 cross bridges	0.990418
N1	Nonpermissive tropomyosin with 1 cross bridge	0.001436
Htrpn__ca_	Concentration of Ca^2+^ bound to high affinity troponin sites (mM)	0.130557
Ltrpn__ca_	Concentration of Ca^2+^ bound to low affinity troponin sites (μM)	0.018066
Nai	Intracellular Na^+^ concentration (mM)	5.853891
Ki	Intracellular K^+^ concentration (mM)	68.3
CKO	Channel closed state	0.992975
CK1	Channel closed state	0.002940
CK2	Channel closed state	0.0019
OK	Channel open state	0.001666
IK	Channel inactivated state	0.000519
Iks__n_	I_Ks_ gate n	0.002189

To simulate isoproterenol stimulation, the L‐type Ca^2+^ current (I_Ca_), transient outward current (I_to_), I_Ks_, I_Kr_ and sarco/endoplasmic reticulum ATPase (SERCA2a) maximal conductances/fluxes were scaled to 1.5, 1.5, 2.0, 2.0, and 2.0, respectively, in accordance with existing literature and based on observed modulations in experimental investigations suggested to simulate the adrenergic response (Colman, 2019; Dibb et al., 2007; Sankaranarayanan et al., 2016, 2017; Stevenson‐Cocks, 2019).

#### 7.2.3 Simulation protocols

##### Action potential repolarization response to increased pacing and adrenergic stimulation

The simulated mLR restitution protocol consisted of pacing at 1, 2, 4, 6, 8, and 10 Hz sequentially, each time preconditioning for a period of 60 s to reach steady‐state conditions before recording the final AP at each frequency (Howlett et al., 2022). Time taken to repolarize to 50% (APD_50_) and 90% (APD_90_) of full AP repolarization was recorded for each pacing frequency. All AP data simulated by the mLR model waere then compared with experimental data and simulated data from the original LR model.

##### Ion Channel responses to increased pacing and adrenergic stimulation

The mLR model was paced sequentially for 60 s at 1, 2, 4, 6, 8, and 10 Hz frequencies under control and adrenergic stimulation conditions and peak I_Ca_, I_to_, I_Ks_, I_Kr,_ and I_K1_ were recorded during the final AP simulated at each frequency. Peak ion current data simulated by the mLR model were compared with the available experimental data where possible as well as data simulated from the original LR model.

##### Sensitivity analysis

To identify current dominance and key current changes underlying APD_50‐90_ responses to changes in pacing and adrenergic stimulation, the restitution protocol was used. The protocol was carried out repeatedly, each time blocking the current flux of a single selected ion channel by 0, 1, 2, 3, 4, 5, 10, 15, 20, 25, 30, 35, 40, 45, 50, 60, 70, 80, 90, and 100%. This process was carried out separately on I_Ca_, I_to_, I_Kr_, I_Ks_ and I_K1_ under control conditions and under conditions of adrenergic stimulation as described previously. In order to ensure complete flux block was achieved during adrenergic stimulation, the flux of upscaled currents (during adrenergic stimulation) required additional block to remove the effects of upscaling by magnitudes of 125% and 150% in I_Ca_ and I_to_ and 125%, 150%, 175%, and 200% in I_Kr_ and I_Ks,_ respectively. After each repetition of the protocol, APD_50_ and APD_90_ were recorded and the changes in APD_50‐90_ were calculated and compared with baseline results. The peak magnitude of APD_50‐90_ change stimulated by current flux block was calculated for each current. Also, the minimum current flux block required to stimulate a 5% change in APD_50‐90_ was also calculated for each current. These two calculations provided the criteria for sensitivity interpretation.

## AUTHOR CONTRIBUTION

Luke A. Howlett: Study conception; data collection; assisted modification of computational model; computational simulations; sensitivity analysis; data analysis; data interpretation; drafting of the original manuscript. Harley Stevenson‐Cocks: Development of computational model; critical revision of manuscript. Michael A. Colman: Development of computational model; critical revision of manuscript. Matthew K. Lancaster: Critical revision of manuscript; study conception. Alan P. Benson: Study conception; development of original computational model; modification of computational model; assisted computational simulations; critical revision of manuscript.

## FUNDING INFORMATION

This work was funded by the British Heart Foundation (grant PG/16/74/32374 to APB), the Medical Research Council UK (Career Development Award MR/V010050/1 to MAC) and the University of Leeds (Demonstrator PhD studentships to MKL and APB).

## CONFLICT OF INTEREST STATEMENT

There were no personal, professional, or financial conflicts influencing this study.

## DISCLAIMER

The work in this manuscript is based on elements of a doctoral thesis (Howlett, 2022).

## Supporting information


Supinfo.

## Data Availability

The data analyzed in this study are available in the journal repository.
