# Supplementary material for: Ionic current changes underlying action potential repolarization responses to physiological pacing and adrenergic stimulation in adult rat ventricular myocytes
Source: Physiol Rep. 2023 Jul 26;11(14):e15766. doi: 10.14814/phy2.15766 (PMC10371833; doi:10.14814/phy2.15766)
Supplement: Supplementary file 1 — Supinfo. [file PHY2-11-e15766-s001.zip › phy215766-sup-0001-Equations Supplement.docx]

**Supplement: The Leeds Rat model**

For this study, we used a combination of two models: the ion channel and membrane potential formulations from the Gattoni model (Gattoni *et al.*, 2016) and the 0D deterministic variant of the Colman calcium-handling model (Colman, 2019). Both models have already undergone parameterisation and validation against experimental data. See the relevant publication for formulations and parameter values.

To ensure close reproduction of action potential and calcium handling characteristics to those observed experimentally, L-type calcium current (*I*_CaL_) steady-state and time constant formulations from the Pandit rat model (Pandit *et al.*, 2001) were used and small adjustments were made to certain parameters (Stevenson-Cocks, 2019). These adjustments are shown in Table 1:

Table 1: Updated parameters in the Leeds Rat (LR) model.

| Parameter | Description | New value |
| --- | --- | --- |
| τ_M,1_ | Time constant of monomer binding | 5 ms |
| τ_Mi,1_ | Time constant of monomer inactivation | 5 ms |
| τ_M,2_ | Time constant of monomer unbinding | 1 ms |
| τ_Mi,2_ | Time constant of monomer de-inactivation | 1 ms |
| *k*_d2d3_ | Rate constant for *d*_2_–*d*_3_ transition | 0.15 ms^-1^ |
| *v*_NSR_ | NSR volume per CRU | 0.1 µm^3^ |
| *N*_LTCC_ | Number of L-type Ca^2+^ channels per dyad | 12 |
| τ_fca_ | Time constant for Ca^2+^ induced inactivation of *I*_Ca,L_ | 9 ms |
| $\bar{\text{Ca}}$ | Ca^2+^ constant for Ca^2+^ induced inactivation of *I*_Ca,L_ | 0.6 µM |
| *g*_leak_ | Max flux rate of *J*_leak_ | 1.284228 × 10^-5^ ms^-1^ |
| *g*_up_ | Max flux rate of *J*_up_ | 0.29481 µM ms^-1^ |

Time constants for RyR monomer binding, unbinding, inactivation and de-inactivation, as well as the rate constant for *d*_2_–*d*_3_ transition, were all reduced to allow quicker recovery of the RyR at faster pacing frequencies associated with rat ventricular myocytes, up to 10 Hz. The network SR (NSR) volume per calcium release unit (CRU) was increased, the number of L-type Ca^2+^ channels per dyad was decreased, the time constant and the Ca^2+^ constant for Ca^2+^ induced inactivation of *I*_CaL_ were decreased, to better reproduce spatial Ca^2+^ dynamics. The conductance of the sarcoplasmic reticulum (SR) Ca^2+^ leak current (*J*_leak_) was reduced to prevent excessive leak of SR Ca^2+^ and maintain a steady SR concentration. The maximal flux rate of SR Ca^2+^ uptake (*J*_up_) was reduced to account for the smaller Ca^2+^ transient magnitudes observed in rat ventricular myocytes compared to larger mammalian myocytes.

For all simulations, a stimulus current with an amplitude of -40 μA/μF for a duration of 2 ms was used.

Please see the Methods section in the manuscript for details of changes made to the combined model specifically for this study.

The complete model, as C code, is available as a supplement to this manuscript.

References:

Gattoni S, Røe ÅT, Frisk M, Louch WE, Niederer SA and Smith NP (2016) The calcium–frequency response in the rat ventricular myocyte: an experimental and modelling study. *Journal of Physiology* **594**, 4193-4224.

Colman MA (2019) Arrhythmia mechanisms and spontaneous calcium release: Bi-directional coupling between re-entrant and focal excitation. *PLoS Computational Biology* **15**, e1007260.

Pandit SV, Clark RB, Giles WR and Demir SS (2001) A mathematical model of action potential heterogeneity in adult rat left ventricular myocytes. *Biophysical Journal* **81**, 3029-3051.

Stevenson-Cocks HJ (2019) Biophysical modelling of rat cardiac electrophysiology and calcium handling. PhD Thesis, University of Leeds.
